# A Simulation Study Comparing Epidemic Dynamics on Exponential Random Graph and Edge-Triangle Configuration Type Contact Network Models

**DOI:** 10.1371/journal.pone.0142181

**Published:** 2015-11-10

**Authors:** David A. Rolls, Peng Wang, Emma McBryde, Philippa Pattison, Garry Robins

**Affiliations:** 1 Melbourne School of Psychological Sciences, University of Melbourne, Melbourne, VIC 3010, Australia; 2 Department of Medicine-RMH, University of Melbourne, Melbourne, VIC 3010, Australia; University of Zaragoza, SPAIN

## Abstract

We compare two broad types of empirically grounded random network models in terms of their abilities to capture both network features and simulated Susceptible-Infected-Recovered (SIR) epidemic dynamics. The types of network models are exponential random graph models (ERGMs) and extensions of the configuration model. We use three kinds of empirical contact networks, chosen to provide both variety and realistic patterns of human contact: a highly clustered network, a bipartite network and a snowball sampled network of a “hidden population”. In the case of the snowball sampled network we present a novel method for fitting an edge-triangle model. In our results, ERGMs consistently capture clustering as well or better than configuration-type models, but the latter models better capture the node degree distribution. Despite the additional computational requirements to fit ERGMs to empirical networks, the use of ERGMs provides only a slight improvement in the ability of the models to recreate epidemic features of the empirical network in simulated SIR epidemics. Generally, SIR epidemic results from using configuration-type models fall between those from a random network model (i.e., an Erdős-Rényi model) and an ERGM. The addition of subgraphs of size four to edge-triangle type models does improve agreement with the empirical network for smaller densities in clustered networks. Additional subgraphs do not make a noticeable difference in our example, although we would expect the ability to model cliques to be helpful for contact networks exhibiting household structure.

## Introduction

It is recognised that contact networks can play an important role in studying and understanding epidemic dynamics [1]. For these purposes, network models are important for at least two reasons. First, researchers may have an empirical network, but want to simulate additional, similar networks to avoid “over-fitting” to the particular observed network. Second, researchers may have no data on empirical contact patterns, so use a model, possibly informed by an empirical or textbook distribution, for example. One family of network models arises from a collection of network research expanding on the standard “configuration model” [2, 3]. An alternative approach uses the family of Exponential Random Graph models [4], combined with simulation of disease transmission. In this paper we try to make some progress in bridging these two network modelling domains in the context of network epidemiology. We do not try to investigate the effects of node degree variation or clustering on epidemic dynamics, per se. (Welch et al. [1] has a nice summary of current results.) Nor do we study the suitability of theoretical solutions, that are asymptotically correct, to finite empirical network sizes. (We recognise some researchers use variations of the configuration model, but then simulate a disease transmission process.) Rather, we are trying to make some progress in understanding which subgraphs are relevant to the study of epidemic dynamics and how the strengths and shortcomings of each modelling domain affect conclusions on epidemic dynamics. A key feature of our work is a focus on empirical contact networks, which therefore have realistic patterns of human contact.

We can list several desirable properties of network models for the study of epidemic dynamics. 1) Key network features (e.g., giant component size, location of phase transitions) can be found without Monte Carlo simulation of an ensemble of networks. 2) They capture “relevant” features of networks (e.g., clustering, homophily), although a sufficient list of such features to study epidemic dynamics is unknown, and which network features are relevant probably varies depending on disease features such as infectivity. 3) Epidemic dynamics are solvable. Key quantities of interest like final size, epidemic duration, and the infectivity threshold for epidemics, are solvable without explicit stochastic simulation of the epidemic (e.g., using a system of ODEs or bond percolation results). 4) Variability in quantities of interest are also solvable. While deterministic models of the infection process provide average (in some sense) values, probabilistic models can provide the variability, and maybe even a distribution.

### Configuration-Type Models

Research based on the configuration model [2, 3] attempts to create a model with these desirable properties. The “standard configuration model” creates networks with a specified degree sequence. In short, a node *i* with degree *d*_*i*_ is allocated *d*_*i*_ “stubs”. Network ties are formed by connecting pairs of stubs at random, thus achieving a network with desired degree sequence. The standard configuration model has proven to be amenable to the study of Susceptible-Infected-Recovered (SIR) epidemics (e.g, [5–7]). For example, a reproduction ratio, *R*_0_, and epidemic threshold analogous to those of compartmental models can be expressed. Define transmissibility [5], *T*, in a homogeneous population with a constant infectious period to be the probability an infected node infects a susceptible neighbour along a single edge. In the early stages of an outbreak on a configuration model network, the expected number of infections caused by a newly infected single node is

$$\begin{array}{c} R_{0}=T\frac{E\left(d^{2}\right)-E\left(d\right)}{E(d)} \end{array}$$ (1)

where *E*(*d*) is the mean node degree and *E*(*d* ^2^) is the mean of the squared node degree (closely related to the variance). For heterogeneous populations and non-constant infectious periods the expression can be generalized to

$$\begin{array}{c} R_{0}=E\left(T\right)\frac{E\left(d^{2}\right)-E\left(d\right)}{E(d)} \end{array}$$

where *E*(*T*) is the average transmissibility over all infectious and susceptible nodes [6, 8]. Using bond percolation arguments, an epidemic is possible only when *R*_0_ > 1 (as for compartmental models), or equivalently the transmissibility exceeds a critical transmissibility [9], *T*_*c*_, given by

$$\begin{array}{c} T_{c}=\frac{E(d)}{E\left(d^{2}\right)-E\left(d\right)}. \end{array}$$

In more general configuration-type networks with triangles and other small-cycle subgraphs, Eq (1) no longer applies. Although the degree distribution will be the same, fewer susceptible nodes will be available for an infection in the early stages of an outbreak, and we would expect the critical transmissibility to be higher, although the extent to which Eq (1) must be modified is the subject of research. Miller [8] considers approximations of *R*_0_ using additional subgraphs, and suggests clustering affects the final epidemic size only if the typical node degrees are small or clustering is very high. The expression for unclustered networks in Eq (1) also serves as a useful guideline and bound.

The “edge-triangle” model is a generalisation of the configuration model that includes clustering [10, 11]. In short, single edges are counted separately from triangles. Each node *i* has *s*_*i*_ single edges (“stubs”) and *t*_*i*_ triangle vertices (“corners”). Networks are formed by connecting pairs of stubs at random and triples of corners at random. The joint distribution of stubs and corners is the analogue to the degree distribution in this model. Volz et al. [12] derive a deterministic solution for an SIR epidemic process on edge-triangle configuration networks. They also derive a solution for final epidemic size of clustered networks that is asymptotically exact for large network sizes using bond percolation approximation methods. Importantly they also extend their results on epidemic dynamics beyond edge-triangle configuration networks to a further generalisation that includes cliques (i.e., fully connected subgraphs) of size 4 and above to model household structure. Cliques of size *n* are included in the network model by analogy with triangles- size *n* clique corners are counted in addition to stubs and triangle corners. We note two of their key results. 1) The global clustering coefficient is not sufficient to determine the full epidemiological impact of clustering. Comparing with a different model [13] such that the two models are calibrated to have the same degree distribution, the same global clustering coefficient and the same disease parameters, epidemic dynamics can be quite different, especially when clustering is extensive. Thus, the *specific nature of the clustering* appears relevant for epidemic dynamics. 2) Clustering slows epidemics and reduces final size, at least for a particular subclass of edge-triangle networks considered (negative binomial degree distribution, tunable fraction of edges in triangles, mean and variance of the degree distribution held constant).

Separately, a simulation study [14] has considered the situations when contact network models, not random mixing, are warranted. When the number of contacts per day is large, or the transmissibility of the infection is high (e.g. mumps, measles, pertussis, chickenpox), random mixing models produce adequate results. On the other hand, when numbers of contacts and transmissibility are low (e.g. MRSA, possibly Ebola), network contact structure is important.

A key weakness of the edge-triangle networks [10, 12] is that triangles (and larger cliques) share a node but not an edge (i.e., no overlapping “motifs”, referred to here as subgraphs). A generalisation that allows arbitrary subgraphs beyond cliques has been proposed [15] for which network features (and importantly, percolation results for epidemic dynamics) remain solvable. For example, the necessary generating functions are provided for edges, triangles, squares, diamonds (a square with diagonal, also known as a 2-triangle), and 4-cliques. The joint distribution for node “roles” is the key distribution of interest. These five subgraphs require six node roles (i.e., two for the diamond). Analogous to the degree sequence is the “role sequence” which is a vector for each node, counting the number of each role in which that node participates. More general subgraphs could be treated similarly, although the necessary generating function may be complicated or difficult to express. A key question is *which subgraphs should be included in the network model for the study of epidemics?*

### Exponential Random Graph Models

Exponential Random Graph models (ERGMs) [4, 16] are a particular class of network models that have proven useful in modelling social networks. As a class of network models, ERGMs offer several advantages. 1) They allow general subgraphs, called “configurations”, without additional machinery. 2) Configurations can share edges as well as nodes. 3) Importantly, they are grounded in hypotheses about social processes underlying network formation. 4) They are parameterised to capture network features relevant to human interaction (e.g., clustering, homophily, social circuit dependence [17, 18]) in a manner that aids interpretation. 5) Very parsimonious models can be shown to capture a large number of network features well. Under a homogeneity assumption whereby all structurally identical subgraphs are equally probable, these models have the form

$$\begin{array}{c} \text{Pr}\left(Y=y\right)=\exp(\sum_{C}\theta_{C}z_{C}\left(y\right))/\kappa , \end{array}$$ (2)

where **Y** = [*Y*_*ij*_] is an *n* × *n* binary matrix of variables denoting whether a tie is present (1) or absent (0) and **y** = [*y*_ij_] denotes a realisation of **Y**. Summation is over all possible subgraph configurations *C*, *θ*_*C*_ is a parameter corresponding to the configuration *C* and is non-zero only if all pairs of networks variables in *C* are conditionally dependent, *z*_*C*_(**y**) = ∏_(*i*, *j*)∈*C*_ *y*_*ij*_ is the network statistic corresponding to the configuration *C* (indicating whether all ties in *C* are observed in **y**), and *κ* is a normalizing constant which ensures Eq (2) describes a proper distribution. The homogeneity assumption means there can be one parameter for each configuration, thus reducing the number of sufficient statistics to specify the model.

As has been pointed out (e.g., [15]), in practice ERGMs are difficult to analyse and can display pathological behaviours that limit their usefulness. The former concern is certainly true in the general case, although there has been some progress [19]. Realistically, the study of both ERGM networks and epidemic dynamics on ERGM networks must rely on stochastic simulation of both the networks and epidemics on the networks. Pathological behaviours, which often make model fitting difficult for the earlier Markov models, are less serious with new social circuit specifications for ERGMs [16, 20]. In addition, several other concerns might be raised. 1) Model fitting currently requires Markov Chain Monte Carlo maximum likelihood estimation (MCMCMLE) techniques that become computationally intensive as the number of nodes and edges grows. This imposes a practical constraint on the size of networks that can be modelled with ERGMs (although the new snowball methods—see below—open possibilities for parameter estimation of large networks). 2) Model fitting has generally assumed the entire network has been observed, which is unrealistic for large networks. However, recent results [21] have relaxed this “whole network” requirement such that a “snowball sample” [22] can be used. An alternative approach for less than whole-network data has also been proposed [23]. 3) Model fitting can be tricky, requiring the “right” collection of configurations to be included to adequately capture network features.

One of the empirical networks used here is actually from a chain-referral sampling technique called “snowball sampling” [22]. It is a network of people who co-inject in Melbourne, Australia [24], an example of a “hidden population” for which snowball sampling (and other chain-referral techniques like “respondent-driven sampling” (RDS) [25]) are often used. There are additional difficulties when fitting a network model to a network sample rather than a network census [26–28]. 1) High degree nodes are more likely to appear in the sample, so their probability will be over-estimated. 2) The samples are sensitive to the initial seed nodes chosen, which in practice may be a convenience sample rather than a random sample. 3) Homophily within the population can lead to some sub-groups being over- or under-represented in the sample, particularly if the set of seed nodes poorly represents the population. While there are techniques to fit ERGMs to snowball sampled data [21], we are not aware of a technique to estimate a network’s node role distribution from a snowball sample. For the purposes of this paper we propose a method to estimate the joint distribution of stubs and corners, thus fitting an edge-triangle model.

## Methods

**Fig 1:**
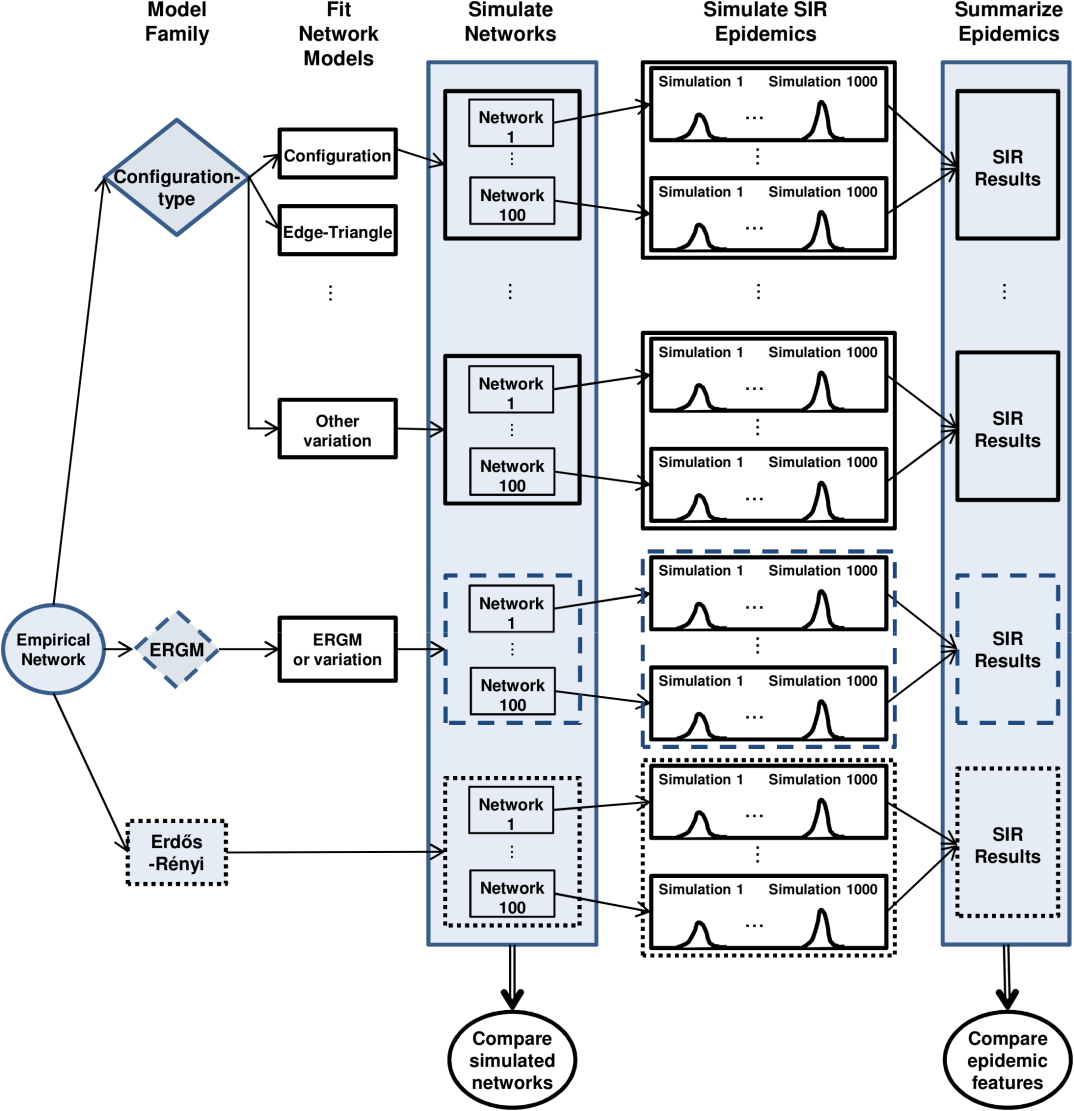
Schematic representation of the study method. For each empirical network we fit a number of network models. For each network model we simulate 100 networks and then simulate 1000 Susceptible-Infected-Recovered (SIR) epidemics per simulated network. Simulated networks are compared with the empirical network in terms of network features and simulated epidemic features.

Our basic approach is to start with an empirical contact network and fit edge-triangle type and ERGM type network models from which we simulate a number of networks. We then simulate SIR epidemics on both the empirical network and simulated networks. We compare simulated networks with the empirical network, and SIR results from using the simulated networks and the empirical network. A schematic representation of the study method is shown in Fig 1.

### Empirical Networks

We use three kinds of empirical contact networks which we further describe below. All networks are assumed symmetric.

#### High School Contact Networks

The high school data [29] describes the cumulative time in close proximity between pairs of people over one day in a U.S. high school. Networks derived from this data provide examples of contact networks relevant to infections where close proximity, but not necessarily physical contact, is important. Such SIR-type infections include corona virus, influenza, norovirus, rhinovirus, varicella and measles. Pertussis (i.e., whooping cough) is an example of a bacterial SIR-type infection (at least in time scales up to years) for which close proximity is relevant.

In total, in this data there are 789 people, comprised of 656 students, 73 teachers, 55 staff, and 5 other individuals. One person has no contacts. By specifying a lower bound *c* for contact duration we can define a network in which a tie represents total contact of at least *c* time units. For this study we consider networks given by 75, 60 and 6 minute durations (referred to as HS75, HS60 and HS6 respectively), which give a range of densities. Clearly the network ties from larger *c* are a subset of the ties from smaller *c*. We use these three networks to illustrate the effects of increasing network density and connectivity. Details of the networks are provided in Table 1. (To confine comparisons of simulated epidemics to similarly sized networks, isolates have been removed from HS75 and HS60 networks.) Mean node degree increases from 2.5 to 57.5 as shorter duration contacts are included. We also show maximal clique sizes. A maximal clique is a clique that cannot be made larger by including an adjacent node (i.e., not a subset of a larger clique). Maximal clique sizes range from 1 to 4 nodes for HS75, to between 1 and 25 nodes for HS6. Loosely, HS75 is a sparse network with small cliques while HS6 is dense with high mean numbers of contacts and large cliques.

**Table 1:** Summary of High School Contact Networks.

Network	Num. Nodes	Num. Isolates	Tie Duration (min.)	Density	Mean Node Degree	Max. Clique Sizes
HS75	538	0	75	0.005	2.48	1–4
HS60	688	0	60	0.008	5.42	1–6
HS6	789	5	6	0.073	57.48	1–25

Property summary of empirical networks from high school data with contact durations at least 75 minutes (HS75), 60 minutes (HS60) and 6 minutes (HS6).

#### Relationships Network

**Fig 2:**
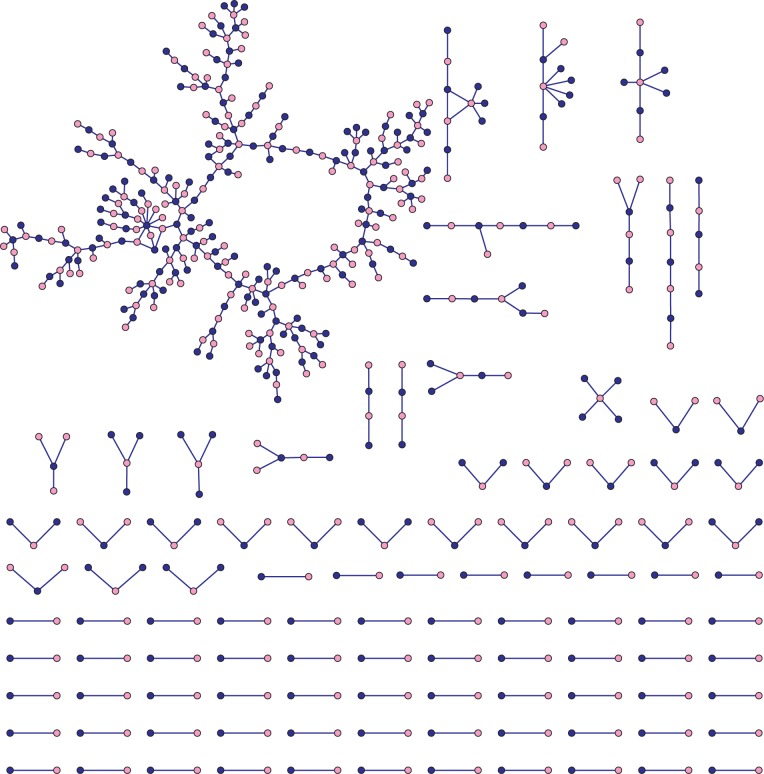
High school relationships network based on the relationships network of Bearman et al. [30]. A network tie indicates a romantic or non-romantic sexual relationship was reported by one of the incident nodes. Gender is denoted by node colour (blue-male, pink-female). This version of the network was manually re-coded starting from Fig 2 of Bearman et al. [30].

The relationships network of Bearman et al. [30] is based on data collected from a U.S. high school in 1994, as part of wave 1 of the Add Health study. If a student reported having a special romantic relationship in the last 18 months, they were asked to describe their three most recent relationships (including any current ones) and up to three individuals with whom they had a non-romantic sexual relationship in the previous 18 months. A network tie represents a romantic, or a non-romantic sexual, relationship between students of the high school or between a student of the high school and another of the feeder middle school. Our version of the network, shown in Fig 2, was manually recoded starting from Fig 2 of Bearman et al. [30] so we have an attribute for gender, but not the additional attributes collected in the study. This contact network is relevant for infections where transmission involves intimate or sexual contact. Such SIR-type infections include cytomegalovirus (CMV) and Epstein-Barr virus (EBV).

In the relationships network there are 573 nodes and 477 edges, including one male-male edge and one female-female edge. If one ignored those two same-sex edges the network would be bipartite, although we illustrate more general models here. Notably, the network has only one triangle, one 9-star and three overlapping 4-cycles. In particular, clustering is not a significant feature of the network.

#### PWID Contact Network

**Fig 3:**
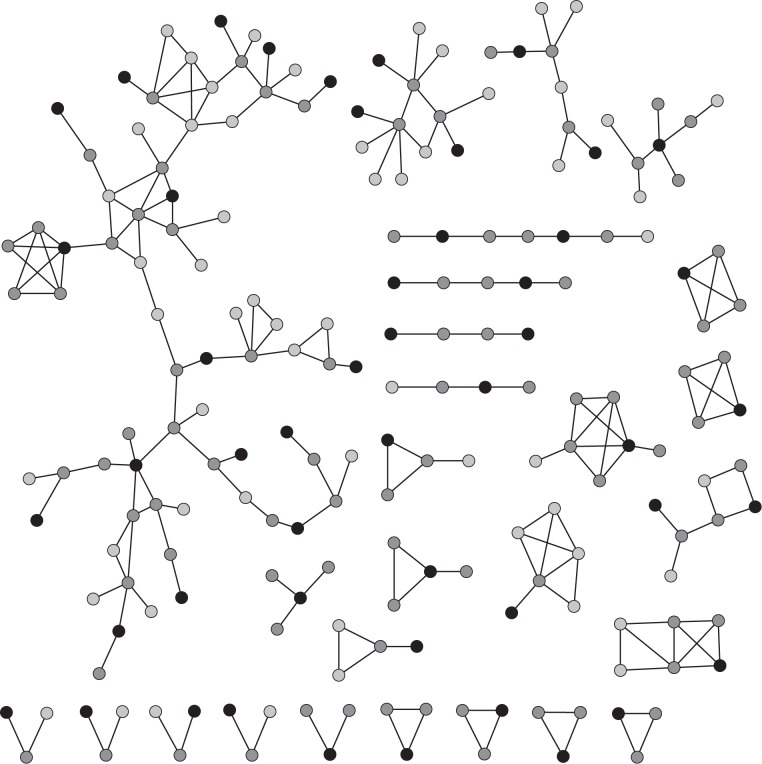
Snowball sample of PWID contact network used for model estimation. Waves are indicated by shade, from wave 0 (black) to wave 2 (light gray).

For this study we use the same empirical PWID network described previously [24] and shown in Fig 3. A tie represents two people have engaged in injecting behaviour at the same place and time within three months prior to interview. The population of interest are in three urban neighbourhoods in Melbourne, Australia. A number of personal details are known for each respondent. Location, age (<25 years or >25 years), gender and injecting frequency (less than daily, at least daily) have previously been included in a contact network model [24] for this data. This contact network is relevant for infections involving blood-to-blood contact. Hepatitis B is an example of an infection that can be approximated as SIR-type.

Importantly, although the data collection used network-based methods, a complete network census was not performed. Rather, we can think of the empirical network as a snowball sample, consisting of seed nodes (“wave 0”, “zone 0”) and nodes in waves *i*, *i* ≥ 1. Assuming every node “remembers” to nominate all its network neighbours, nodes in wave *i*, *i* ≥ 1, are all the nodes nominated by nodes in wave *i* − 1 that are not in wave *i* − 1 themselves. (See Rolls et al. [24] for more details on addressing the effect of missing nominations on the zone structure.)

### Network Models for the High School Networks

For the high school networks we consider several configuration-type models and an exponential random graph model (ERGM). For comparison purposes we also consider an Erdős-Rényi model [31] (sometimes called the *G*(*n*, *p*) model for *n* nodes and edge probability *p*). For each network model we generate a random sample of 100 networks.

#### Configuration-Type Models

**Fig 4:**
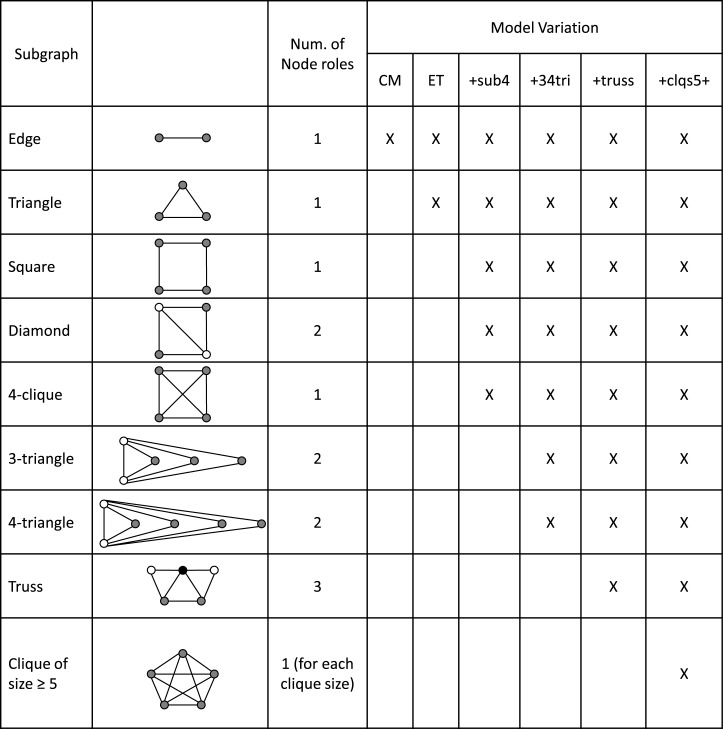
Subgraphs for edge-triangle models. For each subgraph, node roles are distinguished by node colour. The number of node roles for each subgraph is shown in column 3. Inclusion of a subgraph is shown (X) for each of the model variations: standard configuration model (CM), edge-triangle model (ET), variation one with subgraphs of four nodes (+sub4), variation two with 3-triangles and 4-triangles (+34tri), variation three with a “truss”, and variation four with maximal cliques of five or more nodes (+clqs5+).

We consider six configuration-type models: the standard configuration model (CM), the edge-triangle model (ET), a variation of the edge-triangle model that adds subgraphs of size four (+sub4), a variation that then adds 3-triangles and 4-triangles to the first variation (+34tri), a variation that then adds a subgraph with three connected triangles to the second variation (+truss), and a fourth variation that then adds maximal cliques of size five and above to the third variation (+clqs5+). Some of the subgraphs have been proposed elsewhere [12, 15], others help to mitigate the restriction that modelled triangles cannot share edges, and others are motivated by hypotheses on human tie formation. Fig 4 shows the subgraphs, with node colour used to denote the various node roles. The number of node roles is shown in column three. Columns 4–9 show which subgraphs are included in each model variation by the presence of an “X”.

The standard configuration model [2, 3] and the edge-triangle model [12, 15] have been proposed previously. Our first variation of the edge-triangle model adds the subgraphs of four nodes (square, diamond, 4-clique) are described in [15] which also provides the relevant generating functions. The node role sequence is a 6-tuple for each node.

The second variation adds to our first variation the 3-triangles and 4-triangles, which are generalisations of the diamond. They are specific cases of the more general *k*-triangles that have proven useful for ERGM modelling [20]. Theoretically they can arise in connection with *social circuit dependence* [17]. Loosely, social circuit dependence captures the idea that people whose contacts are connected are themselves more likely to be connected.

Our third variation adds to our second variation a subgraph we call a “truss”. This is a five node subgraph with three triangles, a maximum of two sharing any given edge. This experimental subgraph is intended to capture additional clustering where several triangles share edges in a manner not captured by the other subgraphs composed of multiple triangles (i.e., the 3-triangle, the 4-triangle, and the 4-clique). Note that since the diamond is a subgraph of the truss, the node roles for truss membership are assigned with priority over the node roles for diamond membership.

Our fourth variation adds to our third variation maximal cliques of size five and above. Cliques are useful for modelling household structure (e.g., [12]) where close contact between all individuals is assumed. The general assumption of homogeneous mixing between *n* people can be regarded as contact happening in a size *n* clique. Of course, as network density increases, large maximal cliques arise as a consequence of the network having many edges. Since the edge-triangle type configuration models do not allow subgraphs to share edges, high density graphs without explicitly modelled cliques would count many edges as stubs, and a typical network from the model would lack much of this structure.

To model cliques, the maximal cliques of each size *n* are counted in the empirical network. Associated with each size clique is a single node role. That is, in a maximal clique of size *n*, each and every node is connected to *n* − 1 other nodes to form the clique. So if the network has only one occurrence of a size *n* clique, using the node role sequence the same nodes will appear in a size *n* clique in the simulated network. But if there is more than one occurrence of a size *n* clique, size *n* random samples are taken from the collection of all nodes in size *n* cliques.

Earlier work on the edge-triangle [10] and subgraph [15] models describe how to generate networks starting from the node role sequence (or its distribution). They do not say how to count the subgraphs to create the role sequence or estimate its distribution. To model an empirical network this is most critical. Here we describe our method to form the node role sequence by decomposing the empirical network. For example, to fit an edge-triangle model we first count triangle corners at each node, subject to the condition that two triangles cannot share an edge. Thus, once an edge is identified for a triangle, it is removed from use in future triangles. In this sense we “decompose” the empirical network by removing edges as node roles are assigned. Edges not counted as part of a triangle are counted as stubs at their incidence nodes and account for all remaining edges. This approach can be generalised when additional subgraphs are included in the model, as described in S1 Appendix for the decomposition for the model with the largest number of subgraphs (i.e., our fourth variation). Finally we note that how one decomposes the empirical network into non-overlapping subgraphs is not unique. In this study the decomposition generally occurs in decreasing size/complexity, starting with the cliques of at least five nodes (most nodes to fewest nodes) and ending with stubs, as shown in S1 Appendix. Note that node roles for triangles are assigned before node roles for 4-cycles to give the most favourable opportunity to capture clustering. Node roles for the truss are assigned after 3-triangles and 4-cliques, but before diamonds, which are a subgraph of the truss.

To simulate an edge-triangle network, for example, we take the node role sequence and randomly join pairs of stubs and triples of triangle corners as described elsewhere [10, 15]. In particular, we use the observed role sequence instead of fitting a bivariate probability distribution to the number of stubs and corners. Since the quality of that fit would affect our results, using the observed node role sequence provides more controlled comparisons. Joining random stubs and corners can create self-loops and multiple edges. It has been suggested to maintain these ties [15] since excluding them makes theoretical calculations more difficult. Asymptotically, as the number of nodes grows the density of self-loops and multiple edges goes to zero, so they are not such a concern for larger networks. As in [12], we choose to delete them. Self-loops do not add to epidemic dynamics. Multiple edges do not fit within the framework of a binary incidence matrix, and are inconsistent with decomposing the empirical network into subgraphs that do not share edges.

#### Exponential Random Graph Models

To specify an ERGM one provides a parameter for each configuration included in the model. When combined in a model, ERGM parameters represent a balance of competing effects whereby positive (negative) values indicate a tendency to increase (decrease) occurrences of that configuration in the model. The general approach to fitting an ERGM is to select a collection of configurations and use MCMCMLE to estimate parameters while MCMC simulation is used to evaluate model fit and simulate from models. All ERGMs for the high school networks in this study contain five configurations for structure and one configuration for homophily based on person type. The *edge* configuration controls network density. An “isolates” term controls the number of isolates produced by the model. Where the number of isolates in the empirical network is zero, this parameter helps keep the number of isolates small. Note that in the special case where there are no isolates in an empirical network, more negative values of the isolates parameter would work equally well.

Three additional parameters are based on a parsimonious specification of Snijders et al. [20]. These are the *alternating k-star*, *alternating two-path* and *alternating k-triangle* statistics, respectively (see S2 Appendix for definitions). In an equivalent formulation [32], analogous statistics are called the “geometrically weighted degree”, the “geometrically weighted dyadic shared partner” and the “geometrically weighted edgewise shared partner” statistics, respectively. Alternating *k*-stars provide a way to model the node degree distribution. When occurring together in a model, alternating *k*-triangles model triadic closure and alternating two-paths model the prerequisite for triadic closure. They are also useful for modelling social circuit dependence [17, 18]. While it is not clear *a priori* that the simplifying assumptions underlying these statistics should hold for any network, these statistics have proven extremely effective in providing good model fits for many empirical networks.

### Network Models for the Relationships Network

Because the relationships network has 475 male-female edges and just one male-male and one female-female edge, the male-female bipartite network is an important part of the network structure. We consider a bipartite version of the configuration model that maintains the node degree distribution and gender structure of ties (i.e., male-female, male-male, female-female). We also consider an ERGM and a multilevel ERGM which both explicitly capture the gender structure of ties. For comparison purposes only, we also consider two models that ignore the gender structure of ties: the edge-triangle model and a random network model that randomly rearranges the network ties. For each network model we generate a random sample of 100 networks.

#### Configuration-Type Models

For a configuration-type model that considers the gender structure of ties we form the node role sequence for the male-female network and form new networks by taking pairs of stubs with the restriction that each edge is formed by one female and one male stub. This fixes the degree sequence for both the male and female groups, and is analogous to the approach of extending the configuration model to include assortativity by type [33]. Note there are no triangles in the bipartite network so triangle corners are not needed. For the purposes of comparing with other models we explicitly put the two same-sex edges into the simulated networks, at the same incident nodes as in the observed network, but acknowledge these two edges will have a negligible impact on the SIR disease simulations.

#### Exponential Random Graph Models

For the ERGM, instead of using an homophily parameter to force most edges into the bipartite structure, we explicitly fix the presence and absence of same-sex ties so that the bipartite network is the network that is modelled. The latter approach is more computationally efficient and recognises the lack of same-sex ties makes model fitting more difficult. To obtain a model with satisfactory goodness-of-fit, the edges of the 9-star were explicitly fixed also. For this network, the 9-star is not typical of the network and violates the homogeneity assumption that is key to ERGM modelling.

An alternative approach to modelling this network would start with a bipartite ERGM [34–36] and explicitly include (or ignore) the two same-sex edges. Instead, we treat the network as an example of a *multilevel* network and use a multilevel ERGM [37] to model the male-female, female-female and male-male networks together with interactions between the three networks. (Note that the same-sex networks are characterised not just by the presence of one edge each, but also by the *absence* of all other possible same-sex edges.) To simplify the modelling we fix the two same-sex edges and model just the male-female bipartite network and any cross-level interactions between the networks. To obtain a model with satisfactory goodness-of-fit, edges of the 9-star, three overlapping 4-cycles and the one triangle were fixed. (Thus, 17 of 475 edges were fixed.) Finally, we flatten the network to create a unipartite network with nodes that have a binary gender attribute.

### Network Models for the PWID Network

For the PWID network we consider an edge-triangle model, an ERGM, and an Erdős-Rényi model (strictly for comparison). For each network model we generate a random sample of 100 networks. Because the empirical PWID network is really a snowball sample, fitting a network requires some care. Techniques that assume the entire network was observed will be biased. Since a whole network census may be infeasible, fitting to snowball sample data is not an uncommon problem.

Note that when starting from a snowball sample, the size of the population is unknown so this must be determined before networks can be simulated. Previously, an ERGM-based, model-dependent estimate of the size was reported as 524 people [24] and shown to be plausible using independent sources of data. To facilitate comparisons we simulate networks from all three models using 524 nodes.

#### Configuration-Type Models

The edge-triangle model is fit to the data using the technique described in S3 Appendix. In short, we consider six estimators for the node degree distribution, form an estimate for a node’s number of triangle corners conditional on it’s degree, simulate networks and form snowball samples to identify which estimated node degree distribution produces snowball samples most like the observed sample. The choice of an estimator for the node degree distribution is not straightforward and balances effects such as bias from the higher likelihood of including nodes of high degree in a snowball sample, reduced data from excluding particular waves of the snowball, and deviations from simplifying assumptions and approximations. S3 Appendix includes results of a simulation sub-study to assess the node degree estimators.

#### Exponential Random Graph Models

Details on fitting an ERGM to this snowball sample network have appeared elsewhere [24] and use a technique called “conditional estimation” [21]. Note that for the PWID network, only waves 0—2 were used in model fitting. In addition, for technical reasons related to hypotheses about how people limit their risk of infection, only components of size three and above in the empirical network were used in Rolls et al. [24] but we ignore that restriction in simulated networks here to simplify the comparisons. Fig 6 of Rolls et al. [24] shows the largest component from one simulated network using the ERGM.

### SIR Simulations

All outbreak simulations proceed in the same way and are based on nodes being in one of three states: susceptible, infected, or recovered. Parameters for the simulations are shown in Table 2. At time 0 some initial number of seed nodes are explicitly infected. At each time epoch, transmission only occurs along network edges, independently for each infected-susceptible pair, according to a Bernoulli distribution with probability of infection that is fixed for each simulation. Infections are one time unit in duration. Simulations continue until there are no infectious nodes remaining. For each network, 1000 outbreak simulations are performed. As is usual, we choose a minimum outbreak size to define an “epidemic”. Across the three high school networks the minimum outbreak size is about 2.2%, up to rounding. We report the final size, epidemic durations and proportion of simulations yielding epidemics.

**Table 2:** Parameters for SIR Simulations.

	HS75	HS60	HS6	Relationships	PWID
Probability of Transmission	experimental variable				
Number of seeds	1	1	1	10	10
Duration of infection	1	1	1	1	1
“Epidemic” min. size (*R*_*min*_)	12	15	18	40	20

Parameters for SIR simulations.

### Analysis

ERGM parameter estimates, goodness-of-fit and network simulation were performed using PNet [38] (high school networks) or MPNet [35–37] (relationships network). Estimation and simulation for configuration-model type networks was performed using Matlab [39]. Maximal cliques were found using the clique.census command of the SNA package of statnet [40]. All SIR simulations and boxplots were completed using Matlab [39]. Confidence intervals use a Gaussian approximation.

## Results

### High School Contact Networks

Configuration-type models and ERGMs were successfully fit to the HS75 network. Edge-triangle type models here use the node role sequence, which is not parsimonious, so we do not list the counts for the various node roles of each node here. On the other hand, ERGMs often have only a few parameters so the model is easily expressed. Table 3 shows the ERGM specification for a model of the HS75 network. Table 4 shows the goodness-of-fit results (burn-in 10^8^ iterations, 20 000 iterations per network, 4000 networks). Column 1 lists the various statistics that are observed. Column 2 shows the value of the statistic for the empirical network (“Sample”). Columns 3 and 4 show the mean (“Mean”) and sample standard deviation (“Std. Dev.”), respectively, of the statistic over simulated networks. Column 5 shows the “*t*-ratio”, defined as (*X* − *m*)/*s* where *X* is the observed value, and *m* and *s* are the sample mean and sample standard deviation across the simulated networks, respectively. For each parameter, using standard methodology, the model is considered to be a good fit for the empirical network if the *t*-ratio has absolute value less than 0.1 for parameters explicitly in the model (shown in boldface) and less than 2 otherwise. Under a Gaussian distribution approximation these roughly correspond to the middle 8% and 95% of a parameter’s distribution, respectively. Column six shows this requirement expressed as an interval (“Goodness-of-Fit Interval”) given by (*m* − *cs*, *m* + *cs*) where *c* = 0.1 for statistics explicitly fit in the model and *c* = 2 otherwise. For each parameter, the model is a good fit if the observed statistic lies within it’s goodness-of-fit interval. The model itself is a good fit if all the parameters have good fit. The ability of this model specification to closely capture all these features of the empirical network, including ones not explicitly modelled, is strong evidence that a useful model has been specified.

**Table 3:** HS75 Network ERGM Specification.

Statistic	Estimates
Edge	-7.954149
Isolates	-10.994985
Alt. *k*-star (*λ* = 1.50)	0.828329
Alt. *k*-triangle (*λ* = 3.00)	1.392038
Alt. *k*-2-path (*λ* = 3.00)	-0.099633
Homophily: person type	0.259939

ERGM specification for a model of the HS75 network.

**Table 4:** HS75 Network ERGM Goodness-of-fit.

Observed Statistic	Sample	Mean	Std. Dev.	*t*-ratio	Goodness-of-Fit Interval
**Edge**	**666**	**665.497**	**25.841**	**0.019**	**(662.91, 668.08)**
2-star	1615	1598.32	161.321	0.103	(1275.68, 1920.96)
3-star	1483	1420.114	262.421	0.24	(895.27, 1944.96)
4-star	1093	990.192	313.791	0.328	(362.61, 1617.77)
5-star	649	566.198	311.516	0.266	(-56.83, 1189.23)
Triangles	80	78.913	14.606	0.074	(49.70, 108.13)
**Isolates**	**0**	**0.005**	**0.069**	**-0.069**	**(0.00, 0.01)**
2-Triangle	65	55.432	29.701	0.322	(-3.97, 114.83)
Bow tie	57	62.922	42.339	-0.14	(-21.76, 147.60)
4-Cycle	64	43.681	20.232	1.004	(3.22, 84.15)
**Alt. *k*-star (*λ* = 1.50)**	**967.347**	**965.926**	**69.811**	**0.02**	**(958.94, 972.91)**
**Alt. *k*-triangle (*λ* = 3.00)**	**219.741**	**219.328**	**36.8**	**0.011**	**(215.65, 223.01)**
**Alt. *k*-2-path (*λ* = 3.00)**	**1574.074**	**1570.435**	**153.481**	**0.024**	**(1555.09, 1585.78)**
**Homophily: person type**	**507**	**506.628**	**23.016**	**0.016**	**(504.33, 508.93)**
Std. Dev. degree dist.	1.534	1.511	0.082	0.282	(1.35, 1.68)
Skewness degree dist.	1.217	1.114	0.141	0.731	(0.83, 1.40)
Global Clustering Coefficient	0.149	0.147	0.017	0.074	(0.11, 0.18)
Mean Local Clustering	0.11	0.1	0.014	0.723	(0.07, 0.13)
Variance Local Clustering	0.06	0.051	0.008	1.116	(0.04, 0.07)

Goodness-of-fit result for the HS75 network ERGM. Each row shows a different network feature considered. Statistics explicitly modelled are shown in boldface. Empirical values are shown along with the sample mean (“Mean”) and sample standard deviation (“Std. Dev.”) across 4000 simulations. The sample mean and standard deviation are expressed as a *t*-ratio in column five and a goodness-of-fit interval in column six. For all parameters the absolute *t*-ratio is always smaller than 0.1 for parameters explicitly modelled, and smaller than 2 otherwise. Equivalently, the observed statistic always lies within it’s goodness-of-fit interval. Based on standard methodology, the network model re-creates all these features of the empirical network to an acceptable standard.

**Fig 5:**
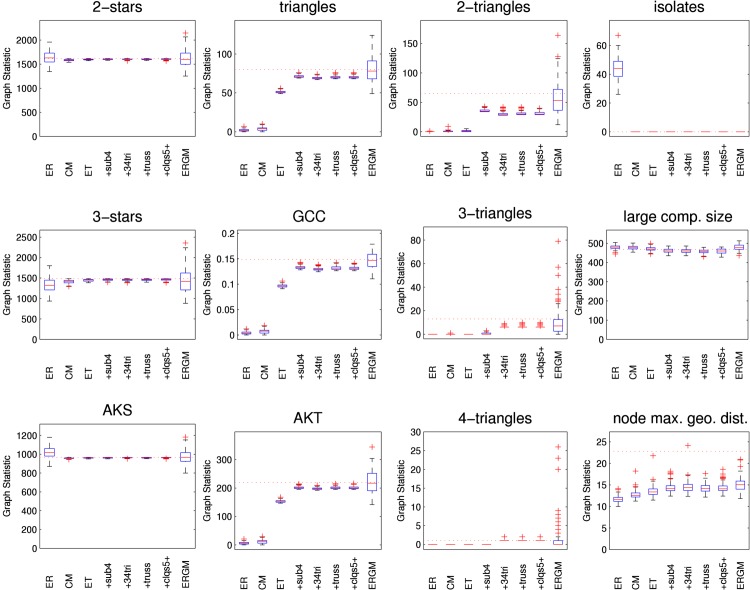
Graph statistics for HS75 network models. Various network statistics shown as boxplots from 100 simulated networks. AKS is alternating *k*-star, GCC is global clustering coefficient, AKT is alternating *k*-triangle, and “node max. geo. dist.” is the mean over all nodes in the largest component of the nodewise maximum geodesic distance. Results are reported for an Erdős-Rényi model (“ER”), the configuration model (“CM”), the edge-triangle model (“ET”) and the four variations which include size four subgraphs (“+sub4”), 3- and 4-triangles (“+34 tri”), trusses (“+truss”), and cliques of size 5 and above (“+clqs5+”), respectively, and an ERGM (“ERGM”). Values from the observed network shown by horizontal dotted lines.

Fig 5 illustrates how well the various models capture various features of the empirical network as boxplots across the simulated networks for each model. As is common in social network analysis, we allow subgraphs to overlap when counting, recognising the idea that a person can be a member of several different groups at once. Boxes show the interquartile range. The central line denotes the median, the whiskers show the range of data not considered outliers, and outliers are shown individually. Where there is no variability the box collapses down to the median. The corresponding value for the empirical network is shown by the horizontal line. Results are reported for an Erdős-Rényi model (“ER”) with the same mean node degree as the empirical network, the standard configuration model (“CM”), an edge-triangle model (“ET”), and the four variations which include size four subgraphs (“+sub4”), 3- and 4-triangles (“+34 tri”), trusses (“+truss”), and cliques of size 5 and above (“+clqs5+”). Each variation listed includes the subgraphs of the models listed before it. Since the largest maximal clique in the HS75 network has only four nodes, the final variation should not show any differences. Finally, we also show results for the ERGM (“ERGM”). (Results for a a random network model that simply randomly rearranges edges [41], sometimes called the *G*(*n*, *m*) model for *n* nodes and *m* edges and referred to hereafter as “ER fix” are indistinguishable from the Erdős-Rényi model and not shown for brevity.)

The three statistics of the first column (2-star, 3-star, alternating *k*-star (AKS)) show how the models are able to capture aspects of the node degree distribution. A different way to assess the agreement of node degree distributions with the empirical network is to use a *χ* ^2^ goodness-of-fit test with 5% significance. With that test, the degree distributions of 38 simulated ERGM networks are rejected. Similarly, 66 networks from the “ER” model and 78 networks from the “ER fix” model are rejected. On the other hand, the configuration-type models maintain the degree sequence. As expected, none of the simulated networks from any of these models are rejected.

Clustering is an important aspect of social networks. The second and third columns of Fig 5 show different aspects of clustering. The second column shows the number of triangles (the key feature of clustering), along with the global clustering coefficient and the alternating *k*-triangle statistic. The third column shows graph statistics for 2-triangles (i.e., diamonds), 3-triangles, and 4-triangles.

The three statistics of the fourth column (number of isolates, size of the largest component, diameter of the largest component (i.e., the maximum geodesic length between any two nodes in the largest component) show aspects of the connectivity of the networks. The *diameter* of the large component is relevant because when the probability of disease transmission is near one, the disease will propagate one hop per time unit. Thus, the large component diameter is an upper bound on the epidemic duration when the probability of disease transmission is near one and nodes are infected and infectious for one time unit. (If there were more than one seed node per component the epidemic duration would typically be less, but the large component diameter would still be indicative.) There is a large difference between the diameter of the large component in the observed network (31) and all the network models. This diameter arises from a connected core and two chains branching out of the core with 8 and 14 nodes each. None of the network models include two of these long chains, so their diameters are smaller.

We can understand the difference in epidemic duration more precisely by noting that every node has a maximum geodesic distance to some other node(s). For probabilities of transmission near one, the distribution of those maxima will govern the distribution of the epidemic duration. Indeed, if nodes are infected and infectious for one time unit the epidemic duration from a randomly chosen seed node will be its maximum geodesic distance, plus one for the seed node infection. For the HS75 network, the mean of the nodewise maximum geodesic distances in the large component is 22.8. For the 468 nodes in the large component, 70 have their maximum to the end of the 8 node chain, and 385 have their maximum to the end of the 14 node chain, 13 are equidistant to both end nodes and three are additional nodes. Thus, these two long chains, especially the 14 node chain, are responsible for the large nodewise maximum geometric distances. Without these large chains, the networks generated by the network models have smaller nodewise maximum geometric distances in general.

The *size* of a network’s large component is relevant because theoretical bond percolation results for epidemic final size arise from a related large component in which the probability of edge formation includes disease transmissibility. A tendency for a model to produce a bigger large component suggests bond percolation results will also predict larger epidemic final size.

**Fig 6:**
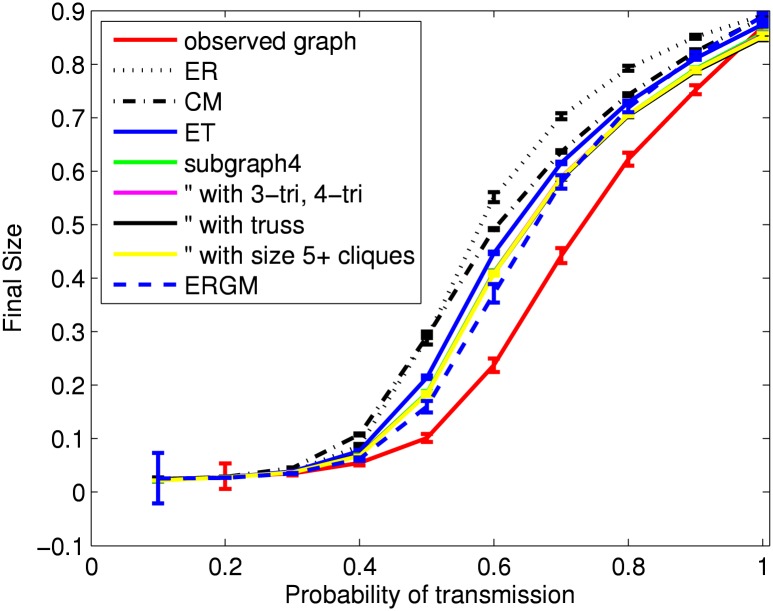
Final size of SIR epidemics using HS75 network models. For each network model, shown are the mean (with 95% confidence intervals) over 100 simulated networks of the mean over all outbreaks from 1000 SIR simulations. For the observed network, shown are the mean (with 95% confidence interval) over all outbreaks from 1000 SIR simulations.

Fig 6 shows results for the estimated final size in the simulated SIR epidemics. For each probability of transmission and network model, results are reported as the mean and 95% confidence interval (across 100 networks) of the mean final size (across 1000 SIR simulations). The similarity in final size across models when the probability of transmission is near one reflects the similarity in large component size.

**Fig 7:**
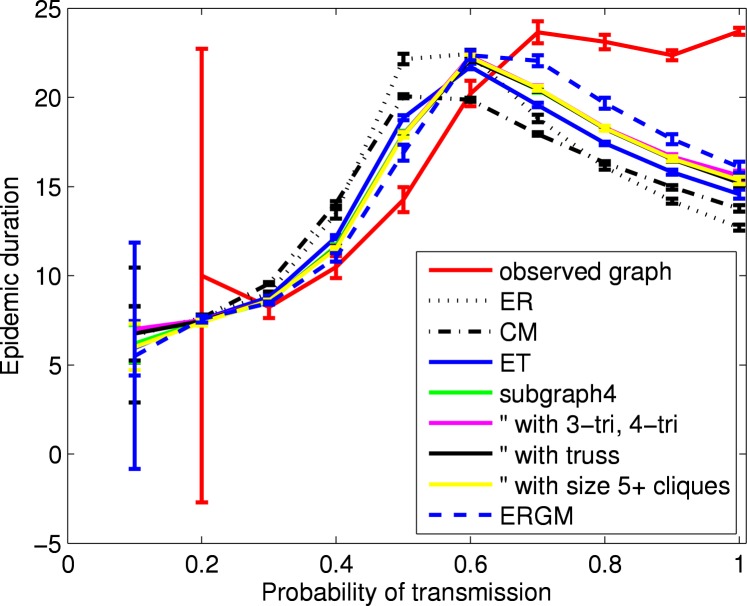
Epidemic duration of SIR epidemics using HS75 network models. For each network model, shown are the mean (with 95% confidence intervals) over 100 simulated networks of the mean over all outbreaks from 1000 SIR simulations. For the observed network, shown are the mean (with 95% confidence interval) over all outbreaks from 1000 SIR simulations. Large (or missing) confidence intervals for small probabilities of transmission arise from a small number of observed outbreaks (typically less than five). The large difference between observed network and the network models for probabilities of transmission larger than 0.7 reflects differences in the large component diameters.

Fig 7 shows similar results for epidemic duration. Note that large (or missing) confidence intervals for small probabilities of transmission arise from small numbers of observed epidemics. (Recall that an epidemic requires a minimum number of infected nodes (*R*_*min*_) as shown in Table 2.) The results for probability of transmission larger than 0.7 (empirical network notably larger than all network models) reflect similar results in large component diameter. More precisely, for the observed network the epidemic duration when the probability of transmission is one is 23.7 (95% CI: 23.5–23.9), which can be understood as 1 time unit for the seed node and then one for each new infection. (Recall the mean of the nodewise maximum geodesic distances in the large component is 22.8.)

Epidemics can vary in how long they take to get started. Miller [8] suggests time shifts to account for these differences before temporal averaging. To further investigate differences across network models, we aligned the start of epidemics by accounting for the variable times to achieve *R*_*min*_. For each simulation we record the time until just before *R*_*min*_ is achieved, and the durations from that time until the peak incidence of the epidemic and until the epidemic is over. We find that across the network models, the mean times until *R*_*min*_ is achieved are indistinguishable from each other and from results for the observed network (not shown for brevity). Further, graphs of the time from *R*_*min*_ to both the epidemic peak and the end of the epidemic resemble Fig 7 but rescaled in the vertical direction (not shown for brevity). Importantly, even after accounting for the variability in the time for an epidemic to get started, epidemics still last longer (on average) on the observed network for probabilities of transmission larger than 0.7. Another quantity of interest, the proportion of simulations with epidemic outbreaks is similar to final size across the network models (not shown for brevity). In particular, it illustrates that for configuration-type networks, outbreaks are less likely to occur when additional clustering is present.

A similar study was performed for the HS60 network which has more clustering. Table 5 shows the model specification for the ERGM. Goodness-of-fit results show excellent agreement with the empirical network, but are not shown for brevity. In *χ* ^2^ goodness-of-fit tests with 5% significance, the degree distribution of 52 simulated ERGM networks are rejected. All 100 networks from the “ER” model are rejected. As expected, no degree distributions from configuration-type networks are rejected.

**Table 5:** HS60 Network ERGM Specification.

Statistic	Estimates
Edge	-8.62622
Isolates	-11.891
Alt. *k*-star (*λ* = 1.50)	1.137897
Alt. *k*-triangle (*λ* = 3.00)	1.250777
Alt. *k*-2-path (*λ* = 3.00)	-0.047
Homophily: person type	0.092378

ERGM specification for a model of the HS60 network.

**Fig 8:**
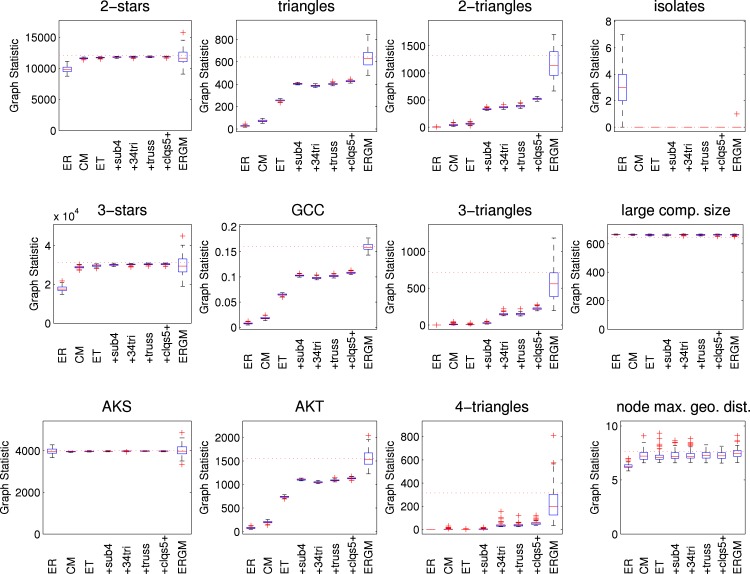
Graph statistics for HS60 network models. Various network statistics shown as boxplots from 100 simulated networks. AKS is alternating *k*-star, GCC is global clustering coefficient, AKT is alternating *k*-triangle, and “node max. geo. dist.” is the mean over all nodes in the largest component of the nodewise maximum geodesic distance. Results are reported for an Erdős-Rényi model (“ER”), the configuration model (“CM”), the edge-triangle model (“ET”) and the four variations which include size four subgraphs (“+sub4”), 3- and 4-triangles (“+34 tri”), trusses (“+truss”), and cliques of size 5 and above (“+clqs5+”), respectively, and an ERGM (“ERGM”). Values from the observed network shown by horizontal dotted lines.

**Fig 9:**
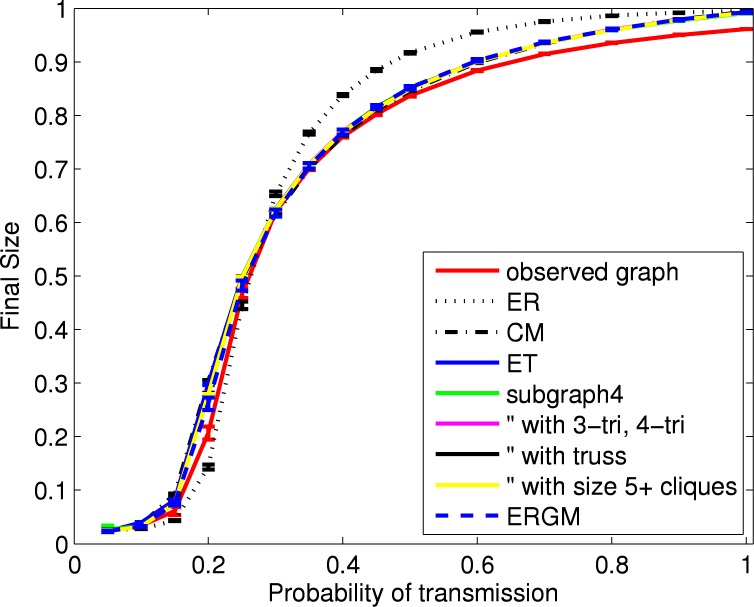
Final size of SIR epidemics using HS60 network models. For each network model, shown are the mean (with 95% confidence intervals) over 100 simulated networks of the mean over all outbreaks from 1000 SIR simulations. For the observed network, shown are the mean (with 95% confidence interval) over all outbreaks from 1000 SIR simulations.

Fig 8 shows results for various graph statistics, and are qualitatively similar to those for the HS75 network. Fig 9 shows results for the final size from the SIR simulations. In contrast to the HS75 results, there is little difference between the configuration-type models, the ERGM and the empirical network. As with the HS75 network, the random networks (“ER”) overestimate the final size for larger probabilities of transmission. The maximum difference in the mean final size is about 8%.

In results for the epidemic duration (not shown for brevity) the configuration-type models are indistinguishable, but do have shorter epidemics than the observed network for probabilities of transmission above 0.2 (e.g., 20.1 vs. 23.3 when the probability of transmission is 0.25). Results for the ERGM are similar, but the differences with the observed network are smaller (e.g., 20.7 vs. 23.3 when the probability of transmission is 0.25). Results for the “ER” model are quite different qualitatively, and show the largest differences with the observed network over a range of probabilities of transmission. Conclusions on the time to *R*_*min*_ and the times from *R*_*min*_ to epidemic peak and epidemic end are qualitatively similar to those for the HS75 network, with the exception of the “ER” model which shows notable differences with the other models for probabilities of transmission below 0.5.

The HS6 network is the densest network modelled here, and has mean node degree of 57. Because edge-triangle type models cannot have overlapping subgraphs, we would expect difficulty for these models to capture network features. In *χ* ^2^ goodness-of-fit tests with 5% significance, the degree distribution of all simulated edge-triangle type networks is rejected. A key reason is surely the number of multiple edges deleted. While the network has 22675 edges, approximately 1100 to 1200 edges are removed because they are multiple edges. A number of graph statistics show little variation across networks. Because of the large density, the edge-triangle type networks are all composed of five isolates and one large component with all the remaining nodes and there is little variation in this large component.

**Fig 10:**
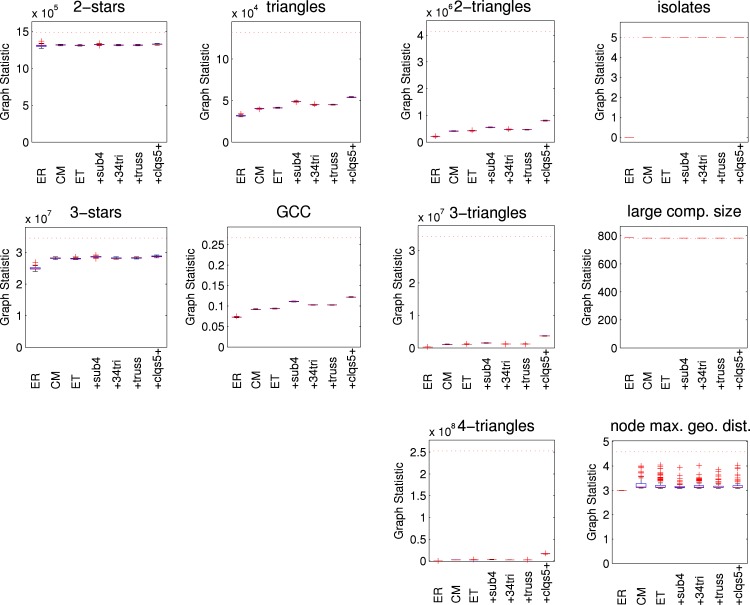
Graph statistics for HS6 network models. Various network statistics shown as boxplots from 100 simulated networks. GCC is global clustering coefficient, and “node max. geo. dist.” is the mean over all nodes in the largest component of the nodewise maximum geodesic distance. Results are reported for an Erdős-Rényi model (“ER”), the configuration model (“CM”), the edge-triangle model (“ET”) and the four variations which include size four subgraphs (“+sub4”), 3- and 4-triangles (“+34 tri”), trusses (“+truss”), and cliques of size 5 and above (“+clqs5+”), respectively. Values from the observed network shown by horizontal dotted lines.

For the ERGM, which is computationally intensive, the large numbers of nodes and edges also make model fitting difficult. In fact, attempts to fit ERGMs were not successful. While fitting a model with a few configurations (e.g., edge + alternating star (*λ* = 1.2) + homophily) was successful in capturing some network features, such models did not sufficiently capture a number of network features not explicitly modelled to a standard common in social network analysis. Fitting models with additional configurations, to improve model fit, appears computationally infeasible. Thus, we cannot report results for an ERGM. Fig 10 shows results for various graph statistics for the edge-triangle type models. We do not report alternating statistics; in the absence of ERGMs with acceptable goodness-of-fit there are no meaningful values of *λ*_*i*_, *i*, = 1, 2, 3.

SIR epidemic results for the HS6 models (not shown for brevity) are qualitatively similar to those for HS60. For example, the final size results are indistinguishable between the configuration-type models, and closely follow those of the empirical network. Results from the random network models (“ER” and “ER fix”) are similar to the empirical network for final sizes between 0% and 65%. Beyond that, these two models overestimate the final size by 5–7%. The epidemic duration results are similar. The maximum difference for the mean epidemic duration between the empirical network and the various edge-triangle models is small (about 2 weeks) when the empirical network predicts 16 weeks duration. On the other hand, between the empirical network and the random networks the difference is about 5 weeks when the empirical network predicts 16 weeks. As with the HS75 and HS60 networks, with the exception of the “ER” model, accounting for the differences in the time to *R*_*min*_ does not reveal any meaningful differences between the models.

### Relationships Network

Table 6 shows the ERGM specification for a model of the relationships network where the 9-star ties and same-sex network structure are fixed. Goodness-of-fit results show excellent agreement with the empirical network, but are not shown for brevity. The positive alternating *k*-star parameter shows a tendency to create stars in the network, and helps capture the degree distribution. The positive isolated edges parameter “IsolateEdges” creates the components of size two. The (relatively) large triangle parameter creates triangles around one of the two same-sex edges. As with the HS75 network, there are no isolates in the empirical network so the isolates parameter serves to keep the number of isolates produced by the model near zero.

**Table 6:** Relationships Network ERGM Specification.

Statistic	Estimates
Edge	-7.4839
Triangle	4.5806
Isolates	-11.5927
IsolateEdges	0.175
Alt. *k*-star (*λ* = 2)	0.5882

ERGM specification for a model of the relationships network.

Table 7 shows the multilevel ERGM specification for a model of the relationships network where the 9-star edges, three overlapping 4-cycle edges, same-sex networks and one triangle are fixed. Of the seven parameters, five are used for modelling the bipartite network. The two alternating *k*-star parameters assist in modelling the degree distributions of male and female nodes separately, while the isolates parameters serve to keep the numbers of male and female isolates near zero. The final two parameters control the interactions between the bipartite network and either the female-female network (2-star FFM) or the male-male network (2-star MMF). These positive 2-star parameters show a tendency for a node in a same-sex relationship to also appear in male-female relationships. Goodness-of-fit results are presented in S4 Appendix and illustrate both the additional degree of modelling detail that is possible, and also the amount of similarity that can be demonstrated, between the empirical and simulated networks when taking a multilevel view.

**Table 7:** Relationships Network Multilevel ERGM Specification.

Statistic	Estimates
Bipartite Edge	-7.4549
Bipartite Isolates (female)	-11.3222
Bipartite Isolates (male)	-10.2588
Bipartite Alt. *k*-star (female; *λ* = 2)	0.9782
Bipartite Alt. *k*-star (male; *λ* = 2)	0.0703
2-Star(FFM)	0.3454
2-Star(MMF)	1.0056

Multilevel ERGM specification for a model of the relationships network. The 2-star effects including same-sex edges show a tendency for a node in a same-sex relationship to also appear in male-female relationships.

**Fig 11:**
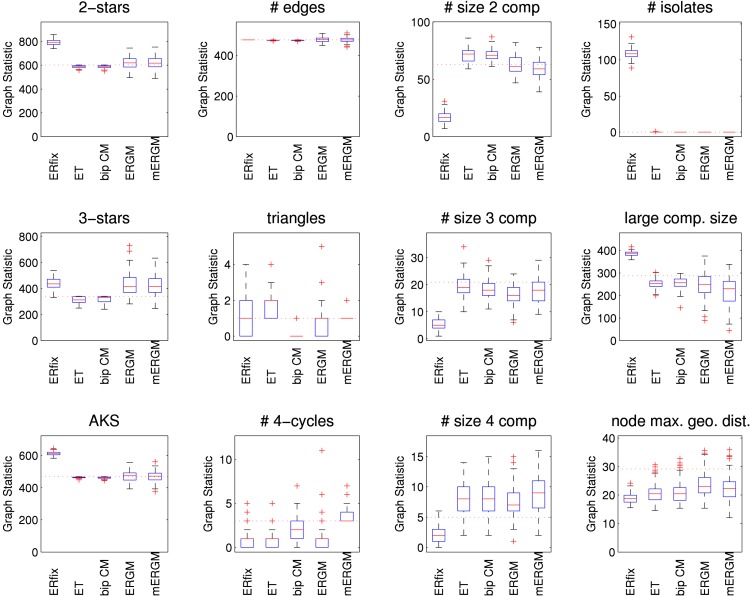
Graph statistics for the high school relationships network models. Various network statistics. AKS is alternating *k*-star and “node max. geo. dist.” is the mean over all nodes in the largest component of the nodewise maximum geodesic distance. Results are reported for a random network model with fixed number of edges (“ER fix”), the edge-triangle model (“ET”) without respecting gender, a bipartite version of the configuration model (“bip CM”), an ERGM (“ERGM”) and a multilevel ERGM (“mERGM”). The last three models include the gender of both nodes of each edge, so they have only two same-sex edges. For the ERGM, the 9-star and the same-sex network tie structure are fixed, exogenous effects. For the multilevel ERGM, the 9-star, the sole triangle, the three overlapping 4-cycles and the same-sex network structure are all fixed. All models use 573 nodes.

Fig 11 shows results for various graph statistics obtained from simulations from the five models: random network with fixed number of edges (“ERfix”), edge-triangle (“ET”), bipartite configuration (“bip CM”), ERGM, and multilevel ERGM (“mERGM”). Because the large number of components is a clear feature of the empirical network, and clustering is not, we focus on quantities related to the degree distribution and connectivity. The numbers of 4-cycles and triangles are also shown for the special role they play in the empirical network to illustrate the ability of the network models to capture these features.

In general, heterogeneity in the genders of relationship pairs (MM,FF,FM) could play a role in the transmission of some diseases, so capturing the numbers of same-sex edges might be important. For the two models that do not respect the gender structure of edges the number of same-sex edges is large (“ERfix”: 239, 95% CI: 237–241; “bip CM”: 235, 95% CI: 232–237). The other three models all have two same-sex edges. In addition, we again used *χ* ^2^ goodness-of-fit tests to check the agreement between the degree distributions of the simulated networks and the empirical network. None of the 100 degree distributions from the two stub models (“ET” and “bip CM”) were rejected at 5% significance while some of the ERGM (24) and multilevel ERGM (37) distributions were rejected. Again, this is despite their seemingly close agreement with the alternating *k*-star statistic. On the other hand, while the 2-stars play the largest role in that statistic, there are clearly important differences in the 3-star statistics (and presumably higher order stars too). While the differences may be acceptable in terms of the *t*-ratio, those differences can still be sufficient for the *χ* ^2^ test to reject. Unsurprisingly, all 100 degree distributions from the “ERfix” model were rejected.

**Fig 12:**
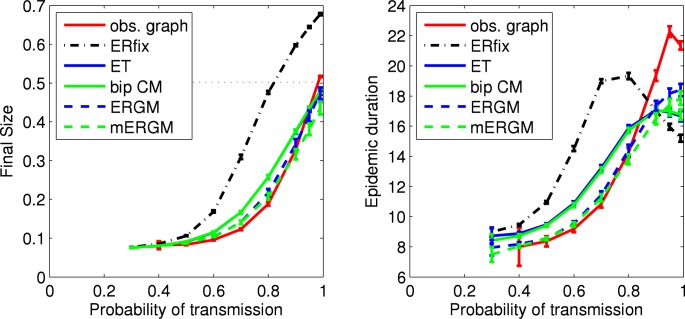
Final size and duration of SIR epidemics using the high school relationships network models. For each network model, shown are the mean (with 95% confidence intervals) over 100 simulated networks of the mean over all outbreaks from 1000 SIR simulations. For the observed network, shown are the mean (with 95% confidence interval) over all outbreaks from 1000 SIR simulations. The dotted horizontal line in the left panel shows the fraction of nodes in the large component of the empirical network. The large number of smaller components effectively limits the final size of an SIR epidemic starting from a limited number of seed nodes.

Fig 12 (left panel) shows results for the final size from simulated SIR epidemics across the five models. To maintain general applicability, the SIR epidemics simulated here do not distinguish between the genders of the two incident nodes, or the direction of transmission. Thus, our SIR results understate the role that nodal attribute heterogeneity might play. So, while results for the edge-triangle and bipartite edge-triangle models are indistinguishable, it is not hard to imagine a more complex disease process for which these two models would give different results. The dotted horizontal line shows the fraction of nodes in the large component of the empirical network. As a result of the large numbers of components, the size of the large component effectively limits the size of an epidemic starting from a limited number of seed nodes. An ordering of the empirical network and network models by decreasing large component size (e.g., “ERfix”, the empirical network, the other four) looks essentially the same as an ordering of the final size when the probability of transmission is one (e.g., “ERfix”, the empirical network, the other four).

Fig 12 (right panel) shows results for the epidemic duration from the simulated SIR epidemics across the five models. An ordering of the empirical network and models by decreasing large component size (e.g., empirical network, “ERGM” and “mERGM”, then down to “ERfix”) looks essentially the same as an ordering of the epidemic duration when the probability of transmission is one (e.g., empirical graph, “mERGM”, “ERGM” then down to “ERfix”).

### PWID Contact Network

Three networks models were fit to the snowball sample PWID network: an edge-triangle model (“ET”), an ERGM (“ERGM”) and an Erdős-Rényi model (“ER”). All networks are simulated with 524 nodes. The Erdős-Rényi model uses the edge probability (*p* = 0.0042) that gives the same mean node degree as the edge-triangle model and empirical network.

**Fig 13:**
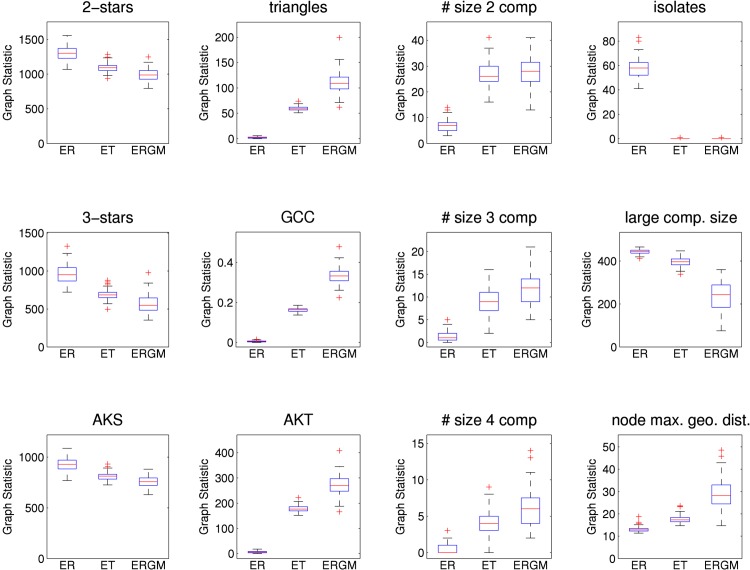
Graph statistics for the PWID contact network. Various network statistics. AKS is alternating *k*-star, AKT is alternating *k*-triangle, GCC is global clustering coefficient, and “node max. geo. dist.” is the mean over all nodes in the largest component of the nodewise maximum geodesic distance. Results are reported for an Erdős-Rényi model (“ER”), the edge-triangle model (“ET”) and an ERGM (“ERGM”). All models use 524 nodes. For snowball sampled network data there is no complete network with which to compare.

**Fig 14:**
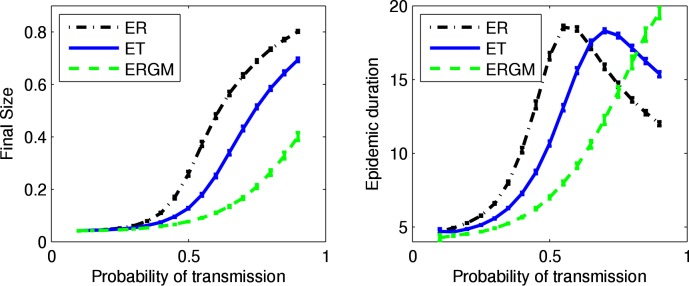
Final size and epidemic duration of SIR epidemics using PWID network models. For each network model, shown are the mean (with 95% confidence intervals) over 100 simulated networks of the mean over all outbreaks from 1000 SIR simulations. For the observed network, shown are the mean (with 95% confidence interval) over all outbreaks from 1000 SIR simulations. For snowball sampled network data there is no complete network with which to compare.

Fig 13 shows boxplots for various graph statistics from 100 network simulations from each of the models. Unsurprisingly, the edge-triangle networks show levels of clustering between the Erdős-Rényi networks (fewest triangles, least clustering) and the ERGM networks (most triangles, most clustering). Fig 14 (left panel) shows results on the final size of SIR epidemics using the PWID network models while Fig 14 (right panel) shows similar results for epidemic duration.

## Discussion

### Degree Distribution

All configuration-type models are able to capture the degree distribution well. Since they are based on the empirical node role sequence, they replicate the degree sequence, except for multiple edges and loops. Looking at the graph statistics for 2-stars, 3-stars and the alternating *k*-star statistic, the ERGM seems to provide a good fit to features of the degree distribution. Indeed, a small *t*-ratio for the alternating *k*-star statistic is often used within social network analysis to argue the network model fits the empirical node degree distribution. Results from the *χ* ^2^ goodness-of-fit tests for both HS75 and HS60 networks illustrate how a traditional statistical test can lead to different results. Indeed, for HS60 there is little difference between the confidence intervals for the alternating *k*-star statistic. Yet, the *χ* ^2^ goodness-of-fit tests rejects none of the networks from the configuration-type models, about half from the ERGM, and all the random networks (“ER”).

The results are somewhat different for the highest density network, for which all the simulated edge-triangle type networks are rejected by *χ* ^2^ goodness-of-fit tests. A key reason is surely the number of multiple edges deleted. While the empirical network has 22675 edges, approximately 1100 to 1200 edges are removed in simulated edge-triangle networks because they are multiple edges. On the other hand, there is very little difference between the models for “2-stars” and “3-stars”. More will be said about these similarities in connection with both clustering and SIR epidemic results.

### Clustering

The number of triangles (the key feature of clustering) illustrates important differences between the models. Unsurprisingly, the random network model (“ER”) produces few triangles. More interesting is that the edge-triangle model also does a poor job of capturing all the triangles. Because of the restriction that triangles in the model cannot share an edge, the edge-triangle model captures only a subset of triangles. Thus, triangles are consistently under-represented in the generated networks in comparison to the empirical network. Further, to capture the node degree sequence, stubs must be used, which makes properties of these networks more closely resemble those of Erdős-Rényi networks.

The addition of size four subgraphs makes a noticeable difference in the HS75 and HS60 networks, but not the HS6 network. This suggests the addition of size four subgraphs are an important addition for capturing network features with edge-triangle type models when both the density is not really large and clustering is not negligible. Additional subgraphs make little difference in the examples considered here. However, household structure is not really a part of the high school network, where cliques might be expected to play an important role. More generally, for the high density HS6 network, as with the star configurations, there are few differences in triangles and clustering between the models. This demonstrates that high density is the main explanation for features in that network.

In our examples, ERGMs do a much better job capturing the number of triangles. The median number of triangles is close to the observed empirical values for both HS75 and HS60 networks, and the interquartile ranges cover the empirical values. (In addition, 95% confidence intervals (not shown for brevity) also cover the empirical values.) Results for the global clustering coefficient are qualitatively similar to those for triangles which illustrates the close connection between those two quantities.

Results for 2-triangles (i.e., diamonds), 3-triangles, 4-triangles and alternating *k*-triangles reflect different aspects of clustering. Since the ERGMs here are parameterised to capture these subgraphs it is not surprising they do a better job modelling these features. Since the edge-triangle model has a restriction preventing triangles from sharing edges, it is not surprising how few of these are captured by the model. It is also interesting to note that the addition of 3- and 4-triangles, and even the truss, to the model with size 4 subgraphs makes little change to the network fit.

### SIR Epidemic Results

Given these differences between configuration-type models and ERGMs (the former models better capture the degree distribution, the latter models better capture clustering) it is unclear which model should best capture epidemic dynamics. For the HS75, HS60 and relationships networks, final size results for ERGMs show closer agreement than the configuration-type models to those of the empirical network. On the other hand, the difference from using a configuration-type model may be small. Results for the HS75 network illustrate some advantage to including subgraphs of size 4, but no advantage for larger subgraphs. After including subgraphs of size 4, differences with using an ERGM may be considered small enough that the configuration-type models are sufficient. As the density increases, the differences in final size from using those subgraphs becomes negligible. We must emphasize again the special role that cliques can play. None of our networks exhibit household structure, whereby a separate network process creates cliques within households which are then loosely connected to each other. We would expect to observe a larger impact from including cliques in those networks.

By comparison, the random network models (“ER”, “ER fix”) show the worst agreement with results from the empirical network. They are indistinguishable from each other in the examples shown here. When the final size is very high (above 60%), final size is consistently overestimated by these models. Below 60% they may either over- or under-estimate, although they under-estimate when the density is not too high. Because these models are not effective in capturing either the degree distribution or clustering, it remains unclear how to attribute differences in epidemic dynamics to these two features.

In the case of high density, highly clustered networks like HS6, there is little variation between the models. Differences from using the empirical network are generally small. Cliques are undoubtedly present, but because the edge density is so high, cliques arise naturally from the large number of edges. This is a different mechanism for clique formation than the one underlying household structure.

Our results are largely consistent with results of Miller [8] which argued in the context of configuration models, that 1) for reasonable networks clustering is only important for final size if node degrees are typically small, and 2) as a consequence, for moderately large transmissibility final size will be unaltered by clustering. Our HS75 network has the smallest node degrees and illustrates some effect of clustering on SIR final size, while HS60 and HS6 have larger node degrees and more clustering but reveal no significant differences in SIR final size across network models with varying amounts of clustering.

Miller [8] argues that although “clustering” is usually taken to mean triangles, it could also refer to other short cycles such as squares and 2-triangles that can also impact the spread of infectious diseases. Some of the network models considered here (especially the ERGM) are parameterised to capture such network features. However, even in a network with mean node degree of 2.5 we found only small differences arising from the ability of a network model to capture such features. A much larger difference is apparent from what might be considered the *absence of clustering* in our HS75 network due to long chains off a highly-connected core. The results on epidemic duration show similarity between edge-triangle models and the ERGM, and all show mean epidemic durations that are too short compared to the observed network. Long chains can slow an epidemic by increasing the number of transmissions required to reach nodes and might also increase the number of cutpoints in the network. Each cutpoint has the potential to interrupt the epidemic by transmission failure. Overall, this suggests there may be advantage in exploring subgraphs that promote longer chains in addition to ones that promote increased clustering.

A common theme in our results is that results for edge-triangle models are between ERGMs and Erdős-Rényi models. This really is not surprising. Edge-triangle models capture some, but not all, of the number of triangles captured by ERGMs, due to overlapping triangles that must become stubs. In addition, edge-triangle models randomly rewire stubs in a manner that removes structure, not unlike the random edges of Erdős-Rényi networks.

Another observation we can make arises from the fact the edge-triangle models have far fewer 3- and 4-triangles than the ERGM, yet show comparable final size results. Our results suggest that while they play an important role in fitting ERGMs, these subgraphs are not playing an important role in determining epidemic dynamics.

### Speed vs. Accuracy

In the networks considered here, SIR epidemic results for ERGMs are generally closer than edge-triangle type models to those for an observed network, although the difference becomes insignificant as density increases. (Of course, all networks become clique-like with high enough density.) Clearly this difference must be balanced by the time spent to develop a model, elapsed time to obtain the model and computational effort to work with a model. In the case of the relationships network for example, in developing the ERGM, 31 models were considered (10 with fixed 9-star and same-sex edges) over the course of a week. Some modeller time was saved by performing estimations overnight. Goodness-of-fit results reported here require 2.5 hours to produce and are an essential step to ensure a useful network model has been found. Twelve earlier goodness-of-fit results (2-3 hours each) ruled out earlier models. In a best case scenario, one could anticipate the need to fix features of the network, and get lucky with the choice of configurations in the model. It is still unlikely to develop the model in under two days. On the other hand, fitting an edge-triangle model can be performed with less specialised expertise and in a negligible amount of time. For the purposes of network epidemiology (not network-based inference which is a separate, excellent domain for ERGMs) these issues must be carefully weighed.

For fitting configuration-type models, the most computationally demanding step is decomposing the empirical network into non-overlapping subgraphs to create the node role sequence. Yet, this is not nearly as demanding as fitting an ERGM. As an example, creating the largest node role sequence for the HS6 network (the largest, densest network used here, with many cliques) required less than eight minutes on a PC. After some effort optimizing the counting algorithms, it likely would perform faster. Because an edge is assigned to only one subgraph, a feature of the decomposition is that once an edge is assigned it can be removed from further consideration. Thus, decomposition of the network becomes less demanding as more edges are assigned to subgraphs. All of this suggests the number of nodes and edges for which the the configuration-type models are useful is at least several orders of magnitude larger than for ERGMs, with the usual provisos on memory and storage limitations for big networks, which are independent of the model.

The decomposition into subgraphs can easily accommodate additional subgraphs. An order for the decomposition is clear when subgraphs are nested- do the larger one first. For subgraphs that have some overlap (e.g., a square and triangle with common edge compared with two squares with common edge) the order for the decomposition is not clear and some kind of choice must be made. It is clear one captures triangle clustering better than the other but that may not be the most important consideration.

There may be an upper-bound on the size of subgraphs that are useful for configuration-type models. As subgraphs increase in size by agglomeration of smaller subgraphs sharing common edges, the number of perimeter edges increases. For example, a 7-node hexagon structure formed from six triangles has six perimeter edges. Inclusion of the hexagon means none of those perimeter edges can be a part of another hexagon, k-triangle or other subgraph. The “cost” for including a hexagon might be to reduce the number of triangles in which some nodes participate.

### Snowball Sampled Data

The use of snowball sampled data instead of a whole network poses special challenges. A key concern is a method to fit edge-triangle type models to such samples. We considered six estimators for the degree distribution that might form the basis of technique to fit edge-triangle type models to a snowball sample. In our example the sample degree distribution estimator seems to produce an estimate for which simulated networks more closely resemble the network sample. Clearly this is not enough to form a universal rule, but does suggest the naive estimator should be considered in similar cases.

There are interesting similarities between the PWID and relationships networks. For example, both have low mean node degree. ERGMs for both produce networks with large cycles and smaller branching chains. There is a similarity to the SIR results too. That is, for fixed transmission probabilities we can form the ranking ERGM < edge-triangle < random network, for both final size and epidemic duration. While this proves nothing, it may be a more common feature of similar networks (low mean node degree, large cycle, branching chains).

Results from fitting the PWID contact network illustrate a general problem that with sampled networks there is no complete empirical network to serve as “truth”. This makes understanding the biases of the various modelling approaches even more important.

### Nodal Attributes

Exponential random graph modelling provides a rich structure to include the role of nodal attributes in network structure and even model both bipartite and multilevel networks. The role of nodal attributes on network structure (i.e., assortative mixing) and the disease transmission process has been developed for the configuration model [33, 42] but we are not aware of similar results for extensions with additional subgraphs. Such results would be a useful development.

### An Interesting Observation on the Relationships Network

We note that Bearman et al. [30] suggests a normative rule is in play to explain the small number of 4-cycles in that network. Further they say that when a 4-cycle term was included in an ERGM, after explicitly accounting for the three 4-cycles occurring through the highly-unusual 9-star, the corresponding 4-cycle parameter was negative. In contrast, we find after explicitly accounting for the 4-cycles and 9-star that small numbers of 4-cycles can be generated in a model (without a need for additional nodal attributes) with acceptable goodness-of-fit that does not explicitly prohibit connected structures larger than triangles. Key differences between the approaches are the use of approximate maximum likelihood estimation and the newer alternating *k*-star statistic here. Bearman et al. [30] used a pseudolikelihood approach and explicitly included 2-stars and 3-stars, which were common approaches at the time.

### Limitations and Future Work

The networks considered here are empirical static networks chosen as illustrative of human contact networks. Our goal here is not to dwell on the particular choices made to create those empirical networks from observational data. We note that Holme [43] studies optimal methods for creating static networks for epidemiological studies from empirical temporal contact data. We also acknowledge there are modes of contact (e.g., physical non-intimate contact, contact within a household structure) not represented by our examples. Clearly the study described here can be extended to such empirical networks in the future.

Advances in the use of technology (e.g., Wifi, Bluetooth, RFID tags) to collect high frequency contact network data (e.g., [29, 44–47]) are creating new opportunities for network epidemiology (i.e., “digital epidemiology” [48]). Part of the opportunity this provides is the exciting ability to study temporal network effects on disease transmission. On the other hand, it is not clear that temporal, rather than static, models will always be necessary. The comparisons by Machens et al. [44] illustrate that a static network model that captures contact durations but not temporal ordering can give SEIR results similar to using full empirical data with a time-varying, individual-based contact network with contact start and end events at 20 s resolution. We adopt the view that the timescales for both epidemic dynamics and contact network dynamics determine the extent to which a static network approximation is useful.

Because our focus is on fitting empirical networks we have not included mathematical network models such as scale-free networks [49] and small-world networks [50]. These models are not parameterised to simultaneously fit the degree distribution and clustering. In the case of scale-free networks, the degree distribution is key to understanding epidemic dynamics [51]. Since the edge-triangle type models can use an empirical node degree sequence, or a node degree distribution, we would expect epidemic dynamics of SIR-type infections would be similar. In the case of small-world networks, the key feature is “shortcuts” that make the typical path lengths shorter than an Erdős-Rényi network. Because the edge-triangle type models randomly re-attach stubs, we would not expect networks from these models to have these shortcuts, and so epidemic dynamics should be more similar to an Erdős-Rényi network.

As a matter of model selection, we would expect better model fit when additional subgraphs are used. We have not attempted to formalize a comparison of these models. We use the empirical node role sequences. If multivariate node role distributions were instead fit to the node role sequences, additional modelling parameters and an additional amount of modelling inaccuracy would be introduced. Some trade-off between model complexity and accuracy would be necessary, but this is beyond the scope of this paper. In many cases researchers will have access to the empirical node roles.

We have focussed on SIR-type infections, but recognise that other types of infections (e.g., SI, SIS) are also important. For the relationships network, most sexually-transmitted diseases would not be modelled as SIR-type. For the PWID network, HIV and hepatitis C are very relevant but would not be modelled as SIR-type. It would be interesting to extend our results to more general infection parameters and types of infections. Edge-based compartmental modelling is a new technique that extends epidemiological results for configuration models to dynamic network models and models where the population has additional heterogeneity (demographic features, multiple types of risk behaviour) and the disease has more complicated history [33, 42]. Unfortunately these methods are not appropriate for SIS-type infections, where the risk of reinfection violates the assumptions underlying simplifications in the calculations. The need for direct simulation, possibly with ERGMs, has not been eliminated for SIS-type infections. Our results could also be extended by looking for differences between the models when a vaccination or treatment is administered. These extensions are left for future work.

We have assumed constant infection duration and both homogeneous infectiousness and susceptibility. Further investigation, in particular to the role of triangles and size four subgraphs in configuration-type models for clustered heterogeneous networks, is left for future work. We note that using theoretical arguments (and demonstrated with a single EpiSimS network) Miller [8] states that heterogeneous infectivity and susceptibility allows short-cycle clustering to play a more significant role in outbreak probability and final size for infections close to the epidemic threshold, but less important for networks with sufficiently large average degree. Certainly, as average degree increases we would expect epidemic results to more closely resemble non-network mixing results.

Our approach to fitting an edge-triangle type model to a snowball sample is novel but ad hoc. We leave as future work to develop this approach to modelling within a statistical framework. In addition, it would be interesting to extend this idea to subgraphs of four nodes, which may require sampling additional snowball waves.

## Supporting Information

S1 Appendix Configuration-type Model Node Decomposition. (PDF)

S2 Appendix ERGM Alternating Graph Statistics. (PDF)

S3 Appendix Fitting an edge-triangle model to snowball sampled data. Describes a novel method to fit an edge-triangle model to snowball sampled data. Six estimators of the node degree distribution are considered. Results from a simulation sub-study are included.
(PDF)

S4 Appendix Multilevel ERGM Goodness of Fit. Tables showing the multilevel ERGM bipartite and cross-level goodness-of-fit results.
(PDF)

S1 Dataset HS75 network files. Files related to the HS75 network. Contains the nodes degrees, graph statistics, and SIR simulation results, for both the empirical HS75 network and simulated networks from various models.
(ZIP)

S2 Dataset HS60 network files. Files related to the HS60 network. Contains the nodes degrees, graph statistics, and SIR simulation results, for both the empirical HS60 network and simulated networks from various models.
(ZIP)

S3 Dataset HS6 network files. Files related to the HS6 network. Contains the nodes degrees, graph statistics, and SIR simulation results, for both the empirical HS6 network and simulated networks from various models.
(ZIP)

S4 Dataset Relationships network files. Files related to the Relationships network. Contains the nodes degrees, graph statistics, and SIR simulation results, for both the empirical Relationships network and simulated networks from various models.
(ZIP)

S5 Dataset PWID network files. Files related to the PWID network. Contains the nodes degrees, graph statistics, and SIR simulation results, for simulated networks from various models fit to the PWID snowball sample.
(ZIP)

S6 Dataset Snowball Sampled Node Degrees for Estimators 1 to 6. Files for evaluating estimators 1 to 6. Node degrees of the snowball-sampled empirical network and snowball-sampled simulated edge-triangle networks fit using estimators one to six in the paper.
(ZIP)
