# Supplementary material for: A Simulation Study Comparing Epidemic Dynamics on Exponential Random Graph and Edge-Triangle Configuration Type Contact Network Models
Source: PLoS One. 2015 Nov 10;10(11):e0142181. doi: 10.1371/journal.pone.0142181 (PMC4640514; doi:10.1371/journal.pone.0142181)
Supplement: S1 Appendix — (PDF) [file pone.0142181.s001.pdf]

S1 Appendix:
Configuration-type Model Node Decomposition of Empirical Networks

The following steps describe our method for decomposing the high school network into node roles for the model that includes maximal cliques of five nodes or more (+clqs5+). Other subgraphs in the model are the 3- and 4-triangles, the "truss", 4-clique, the "diamond" (a 2-triangle), the square (a 2-two-path), triangles, and stubs. Decomposition of models without some of these subgraphs simply skips the appropriate step(s). Notice that node roles for triangles are assigned *before* node roles for 4-cycles to give the most favourable opportunity to capture clustering.

This method assumes the network is unipartite and that a full network census is available. Extensions to bipartite networks and snowball sampled networks are not discussed here.

1. Assign nodes to maximal cliques and remove used edges as unavailable for future use.
2. Identify all two-paths as an aid to finding k-triangles.
3. Count the number of triangles (M_i) sharing each edge *i*.
   While the maximum of M_i over the network is at least 4:
   i. pick a 4-triangle,
   ii. assign node role to 4-triangle,
   iii. remove used edges as unavailable for future use,
   iv. update two-paths and M_i,
   v. repeat step 3 on remaining network.
4. Count the number of triangles (M_i) sharing each edge *i*.
   While the maximum of M_i over the network is at least 3:
   i. pick a 3-triangle,
   ii. assign node roles to 3-triangle,
   iii. remove used edges as unavailable for future use,
   iv. update two-paths and M,
   v. repeat step 4 on remaining network.
5. Count the number of triangles (M_i) sharing each edge *i*.
   While the maximum of M_i over the network is at least 2:
   i. pick a 2-triangle,
   ii. if a 4-clique:
      a. assign node roles to 4-clique,
      b. remove used edges as unavailable for future use,
      c. update two-paths and M_i,
   iii. if not a 4-clique but a truss:
      a. assign node roles to truss,
      b. remove used edges as unavailable for future use,
      c. update two-paths and M_i,
   iv. if not a 4-clique or a truss:
      a. assign node roles to 2-triangle,
      b. remove used edges as unavailable for future use,
      c. update two-paths and M_i,
   v. repeat step 5 on remaining network.
6. Count the number of triangles (M_i) sharing each edge *i*.
   While triangles are present:
   i. pick a triangle,
   ii. assign node role for triangle corners,

iii. remove used edges as unavailable for future use,
iv. update two-paths and M_i,
v. repeat step 6 on remaining network.

7. Find a 4-cycle
    i. assign node role for 4-cycle,
    ii. remove used edges as unavailable for future use,
    iii. update two-paths
    iv. repeat step 7 on remaining network.
8. Assign remaining edges to stubs.
