# Supplementary material for: A Simulation Study Comparing Epidemic Dynamics on Exponential Random Graph and Edge-Triangle Configuration Type Contact Network Models
Source: PLoS One. 2015 Nov 10;10(11):e0142181. doi: 10.1371/journal.pone.0142181 (PMC4640514; doi:10.1371/journal.pone.0142181)
Supplement: S2 Appendix — (PDF) [file pone.0142181.s002.pdf]

S2 Appendix: ERGM Alternating Graph Statistics

Define a k-star to be a node with k incident edges. Define a k-2-path to be a subgraph with two nodes i and j and a set of k paths of length 2 through k distinct intermediate nodes. Define a k-triangle to be a subgraph of two connected nodes i and j and a set of k paths of length 2 through k distinct nodes. Let σ_k, ν_k and τ_k be the model parameters for the k-star, k-2-path and k-triangle configurations, respectively. While this formulation would suggest a large number of parameters, the following geometric weighting greatly reduces their number:

$$\begin{aligned}\sigma_{k+1} &= -\sigma_k/\lambda_1,\ k \geq 2\\ \nu_{k+1} &= -\nu_k/\lambda_2,\ k \geq 3 \text{ and}\\ \tau_{k+1} &= -\tau_k/\lambda_3,\ k \geq 3\end{aligned}$$

for fixed constant weights $\lambda_i \geq 1$, (often taken to be 2), $i = 1, 2, 3$. Under these assumptions single statistics

$$\begin{aligned}S^{\lambda_1}(\mathbf{y}) &= \sum_{k=2}^{n-1} (-1)^k S_k(\mathbf{y})/\lambda_1^{k-2},\\ U^{\lambda_2}(\mathbf{y}) &= U_1(\mathbf{y}) - \frac{2U_2(\mathbf{y})}{\lambda_2} + \sum_{k=3}^{n-2} \left(\frac{-1}{\lambda_2}\right)^{k-1} U_k(\mathbf{y}), \text{ and}\\ T^{\lambda_3}(\mathbf{y}) &= 3T_1(\mathbf{y}) + \sum_{k=1}^{n-3} \left(\frac{-1}{\lambda_3}\right)^{k} T_{k+1}(\mathbf{y})\end{aligned}$$

can be expressed [1], where $S_k(\mathbf{y})$, $U_k(\mathbf{y})$ and $T_k(\mathbf{y})$ are the number of k-stars, k-2-paths and k-triangles in network $\mathbf{y}$, respectively [1]. These single statistics are called the *alternating k-star*, *alternating two-path* and *alternating k-triangle* statistics, respectively.

References

[1] Robins G, Pattison P, Wang P. Closure, connectivity and degree distributions: Exponential random graph (p*) models for directed social networks. Social Networks. 2009 May;31(2):105–117. Available from: `http://dx.doi.org/10.1016/j.socnet.2008.10.006`.
