# Supplementary material for: A Simulation Study Comparing Epidemic Dynamics on Exponential Random Graph and Edge-Triangle Configuration Type Contact Network Models
Source: PLoS One. 2015 Nov 10;10(11):e0142181. doi: 10.1371/journal.pone.0142181 (PMC4640514; doi:10.1371/journal.pone.0142181)
Supplement: S3 Appendix — Describes a novel method to fit an edge-triangle model to snowball sampled data. Six estimators of the node degree distribution are considered. Results from a simulation sub-study are included. (PDF) [file pone.0142181.s003.pdf]

S3 Appendix: Fitting an edge-triangle model to snowball sampled data

We describe here a novel method to fit an edge-triangle model to snowball sample data. Let D_u, S_u and T_u be the node degree, number of stubs and triangle corners at node u from an observed snowball sample. Without additional node roles we must have $D_u = S_u + 2T_u$. Our approach is to use an estimator $\hat{p}(d)$ of the network degree distribution

$$p(d) = P(D_u = d),\ d = 0, \ldots, M$$

where M is the maximum node degree in the network, and an estimator $\hat{p}_{j|d}$ of the conditional triangle corner distribution

$$p_{j|d} = P(T_u = j | D_u = d).$$

We use a distribution conditional on node degree because empirical networks have been shown to exhibit negative correlation between a node's degree and its number of incident triangles [1].

To simulate an edge-triangle network with N nodes we perform the following steps.

1. Simulate a length N random sequence of node degrees $\{D_u\}$ from the distribution $\hat{p}(d)$.
2. Randomly assign a number of triangle corners T_u to each node u according to its degree D_u and the conditional distribution $\{p_{j|D_u}\}$.
3. For each node u, assign the number of stubs S_u according to $S_u = D_u - 2T_u$.
4. Form the network by connecting stubs to stubs and corners stubs to corner stubs.
5. Remove self-loops and multiple edges.

We consider six estimators for the degree distribution and evaluate each in the same way, using the following four steps. (Note that the empirical network is a snowball sample from 49 seed nodes, and waves zero to two.)

1. For a size N network simulate a length N random sample of node degrees $\{D_1, \ldots, D_N\}$.
2. Use the steps above to create the node role sequence (S_u, T_U), $u = 1, \ldots, N$ and create the network.
3. Choose 49 nodes at random as wave 0 and construct a two-wave snowball sample.
4. Repeat steps 1–3 to simulate 100 independent snowball samples from size N networks.

If the hypothesized degree distribution is a reasonable model to generate the simulated networks, the sample degree distribution of the observed snowball sample should resemble the estimated degree distribution from the 100 simulated snowball samples. This can be tested with a χ^2 goodness-of-fit test, for example.

Let $q_A(d)$ be the empirical probability a node in set A has degree d

$$q_A(d) = N_A^{-1} \sum_{u \in A} I_{[D_u = d]}$$

where N_A is the number of nodes in set A. Estimator one uses the empirical probabilities from only the seed nodes

$$\hat{p}(d) = q_A(d),\ A = \{u : W_u = 0\}.$$

If the seed nodes were an independent and identically distributed (i.i.d.) sample from the population, A would be a random sample and this could produce a useful distribution.

Estimator two uses the empirical probabilities from waves 1 and 2 only:

$$\hat{p}(d) = q_A(d),\ A = \{u : W_u \in \{1, 2\}\}.$$

As suggested by Salganik and Heckathorn [2], we disregard the seed nodes for the reason they have been selected by a different, unknown mechanism.

Estimator three uses the empirical probabilities from nodes in waves 0, 1 and 2:

$$\hat{p}(d) = q_A(d),\ A = \{u : W_u \in \{0, 1, 2\}\}. \tag{1}$$

On the one hand we would expect this to be a biased estimate of the degree distribution due to the snowball nature of the sample. On the other hand, if the sample is a large fraction of the population, the potential bias may be small.

Estimator four is the degree distribution estimator

$$\hat{p}(d) = \left(\sum_{j=1}^{\max\{D_u : u \in A\}} j^{-1} q_A(j) \right)^{-1} d^{-1} q_A(d),\ A = \{u : W_u \in \{1, 2\}\}$$

of Salganik and Heckathorn [2]. It is based on the assumption the probability a node appears in the sample is proportional to its degree (so $d\,p(d) \propto q_A(d)$). As with estimator two, we disregard nodes in wave 0.

Estimator five arises from the Volz-Heckathorn RDS estimator [3]

$$\begin{aligned}\hat{p}(d) &= \left(\sum_{u \in A} \frac{1}{D_u} \right)^{-1} \sum_{\substack{u \in A: \\ D_u = d}} \frac{1}{D_u} \\ &= \left(\sum_{u \in A} \frac{1}{D_u} \right)^{-1} N_A q_A(d) \frac{1}{d}\end{aligned}$$

where $A = \{u : W_u \in \{1, 2\}\}$. This estimator also arises from assuming the probability a node is sampled is proportional to its degree.

Finally, estimator six arises from making simplifying approximations to the probability a node is included in the sample [1] so the estimator takes the form

$$\hat{p}(d) = K \frac{q_A(d)}{1 - (1 - N_A/N)^d},\ A = \{u : W_u \in \{0, 1, 2\}\}$$

where K is a constant to ensure probabilities sum to one. This estimator requires knowledge of the network size N which is often unknown. In our case we use the estimated size 524.

Our estimator of the conditional triangle corner distribution is simply the sample conditional triangle corner distribution

$$p_{j|d} = N_A^{-1} \sum_{u \in A} I_{[T_j = j | D_u = d]},\ A = \{u : W_u = \{0, 1\}\}.$$

We disregard the second wave because triangle nodes in wave 2 could have edges to nodes in wave 3 which is beyond the sample. Thus, the counts of triangle corners in wave 2 will be too small, with an unknown effect on the distribution estimate.

For the edge-triangle model, Table 1 shows how well each of the six estimators captures the degree distribution. Column two shows results from using each of the six estimated degree distributions to simulated edge-triangle networks. Because the population network is unknown we compare the observed

snowball sample with a snowball sample of the simulated edge-triangle network with the same number of seed nodes. Shown are the number of networks rejected (out of 100) using a χ^2 goodness-of-fit test at 5% significance for each estimator. This shows that amongst the six estimators considered, estimator three is the only one which rejects at the level of the test. This is evidence that the degree distribution produced using estimator three is consistent with the empirical network and suggests it is the best choice of the six to model the contact network.

To verify this result, we conducted a sub-study by simulating 100 networks with 524 nodes using our ERGM model of the PWID network to serve as "known truth". From each network, we formed 100 snowball samples by using 100 independent seed sets of 49 nodes (so 10,000 samples in total). For each sample we used a χ^2 goodness-of-fit test at 5% significance to compare the estimated degree distribution of the sample with the degree distribution of the corresponding network. Column three of Table 1 shows the mean number of samples rejected (with 95% confidence intervals), calculated as the mean over 100 samples per network, then the mean over 100 networks. We expect rejection rates higher than the level of the test because the samples are only a fraction of the data. Estimator three (the sample degree distribution on waves 0, 1 and 2) does the best at capturing the degree distribution of the population. Although this estimator will be biased by the higher likelihood of including higher degree nodes, it seems (in this case) this effect on producing good distribution estimations is small in comparison to the effect from excluding waves, the inaccuracies from the assumptions of estimator five, or the approximations of estimator six. Thus, we use estimator three to generate edge-triangle type networks.

Table 1: χ^2 **Results for Node Degree Distribution Estimators**

Estimator	Number of Edge-triangle Networks Rejected	Sub-study Mean Number Rejected
1	13	92 (95% CI: 91.3-92.6)
2	22	62 (95% CI: 59.8-64.1)
3	6	46 (95% CI: 43.4-48.1)
4	16	100 (95% CI: 100.0-100.0)
5	100	100 (95% CI: 100.0-100.0)
6	81	100 (95% CI: 99.4-99.7)

Column one identifies which estimator was studied. Column two shows the number of simulated edge-triangle networks rejected (of 100) at 5% significance using degree distribution estimators 1–6. For each network the sample degree distribution is compared with the sample degree distribution of the observed snowball sample using a χ^2 goodness-of-fit test. Column three shows results from a simulation sub-study using 100 simulated ERGMs and 100 snowball samples per ERGM network (so 10,000 samples in total). For each snowball sample, the estimated degree distribution is compared with the degree distribution of the population network ("known truth") using a χ^2 goodness-of-fit test. Both results suggest estimator three generates a degree distribution most like that of the population network.

References

[1] Illenberger J, Flötteröd G. Estimating network properties from snowball sampled data. Social Networks. 2012;34(4):701 – 711. Available from: http://www.sciencedirect.com/science/article/pii/S0378873312000548.

[2] Salganik MJ, Heckathorn DD. Sampling and Estimation in Hidden Populations Using Respondent-Driven Sampling. Sociological Methodology. 2004 Dec;34(1):193–240.

[3] Volz E, Heckathorn DD. Probability based estimation theory for respondent driven sampling. Journal of Official Statistics. 2008;24(1):79–97.
