# Supplementary material for: A Simulation Study Comparing Epidemic Dynamics on Exponential Random Graph and Edge-Triangle Configuration Type Contact Network Models
Source: PLoS One. 2015 Nov 10;10(11):e0142181. doi: 10.1371/journal.pone.0142181 (PMC4640514; doi:10.1371/journal.pone.0142181)
Supplement: S4 Appendix — Tables showing the multilevel ERGM bipartite and cross-level goodness-of-fit results. (PDF) [file pone.0142181.s004.pdf]

S4 Appendix:
Multilevel ERGM Goodness of Fit

Tables S4.1 and S4.2 show the goodness-of-fit results (burn-in 1,000,000 iterations, 1,000,000 iterations per graph, 1000 graphs) for the multilevel exponential random graph model (ERGM) for the relationships network. Results were generated using MPNet [1-3]. Table S4.1 shows the results for just the bipartite network. Column 1 lists the various statistics that are evaluated. Column 2 shows the value of the statistic for the empirical network ("Sample"). Columns 3 and 4 show the mean ("Mean") and sample standard deviation ("Std. Dev."), respectively, of the statistic over simulated networks. Column 5 shows the "*t*-ratio", defined as (observed value-mean value)/standard deviation. For each parameter, using standard methodology, the model is a good fit for the empirical network if the "*t*-ratio" has absolute value less than 0.1 for parameters explicitly in the model (shown in boldface) and less than 2 otherwise.

Table S4.2 shows the additional goodness-of-fit results arising from treating the network as multilevel. In particular, results for female-bipartite, male-bipartite, and female-bipartite-male network interactions are shown. All statistics are fit to an acceptable standard. Because the same-sex networks are fixed, graph statistics without bipartite edges show zero variation. The ability of this model specification to closely capture all these features of the empirical network, including ones not explicitly modelled, is strong evidence that a useful model has been specified.

Table S4.1: Relationships Network Multilevel ERGM Bipartite Goodness-of-fit

Statistic	Sample	Mean	Std. Dev.	*t*-ratio
**Bipartite Edge**	**475**	**474.841**	**12.765**	**0.012**
Bipartite 2-Star (female)	307	312.222	30.721	-0.17
Bipartite 2-Star (male)	283	293.157	26.154	-0.388
Bipartite 3-Star (female)	160	184.229	44.815	-0.541
Bipartite 3-Star (male)	168	220.468	53.499	-0.981
Bipartite 3-Path	709	808.505	130.829	-0.761
Bipartite 4-Cycle	3	3.774	1.004	-0.771
Bipartite Edge-cycle (female)	4	12.738	7.271	-1.202
Bipartite Edge-cycle (male)	24	34.193	9.374	-1.087
Bipartite Isolates (female)	**0**	**0.008**	**0.089**	**-0.09**
Bipartite Isolates (male)	**0**	**0.006**	**0.077**	**-0.078**
Bipartite Alt. *k*-star (female; λ=2)	**239.3125**	**239.0828**	**18.307**	**0.013**
Bipartite Alt. *k*-star (male; λ=2)	**222.6328**	**222.5978**	**17.013**	**0.002**
Bipartite Alt. cycle (female; λ=2)	305.75	310.6015	30.626	-0.158
Bipartite Alt. cycle (male; λ=2)	281.5	291.275	26.059	-0.375
Bipartite Alt Edge-cycle (female; λ=2)	4	8.2503	3.783	-1.124
Bipartite Alt Edge-cycle (male; λ=2)	8.9531	11.9311	2.942	-1.012

Table S4.2 Relationships Network Multilevel ERGM Cross-level Goodness-of-fit

Statistic	Sample	Mean	Std. Dev.	*t*-ratio
**2Star (FFM)**	**6**	**6.182**	**2.443**	**-0.075**
Alt. *k*-star (female; λ=2) with one MF edge	0	0	0	N/A
Alt. *k*-star (bipartite; λ=2) with one FF edge	5.25	5.9453	4.058	-0.171
Female with alt. *k*-star female (λ=2), alt. *k*-star male (λ=2)	242.6875	242.2922	18.361	0.022
Triangle (FMF)	1	1.016	0.125	-0.128
3Path (MFFM)	1	1.008	0.063	-0.128
Alt. *k*-triangle (FMF; λ=2)	8	9.582	7.971	-0.198
EXTA	0	0	0	N/A
**2Star (MMF)**	**6**	**5.933**	**2.298**	**0.029**
Alt. *k*-star (male; λ=2) with one MF edge	0	0	0	N/A
Alt. *k*-star (bipartite; λ=2) with one MM edge	5	5.4942	3.768	-0.131
Male with alt. *k*-star female (λ=2), alt. *k*-star male (λ=2)	226.1328	225.7837	17.12	0.02
Triangle (MFM)	0	0.029	0.174	-0.167
3Path (FMMF)	0	0.0285	0.169	-0.169
Alt. *k*-triangle (MFM; λ=2)	9	8.897	7.325	0.014
EXTB	0	0	0	N/A
3Path (FFMM)	0	0.068	0.26	-0.262
4Cycle (FFMM)	0	0	0	N/A
Alt. *k*-star (female; λ=2) joined to alt. *k*-star (male; λ=2)	0	0	0	N/A
Std. Dev. degree dist. (female-female)	0.084	0.084	0	N/A
Skewness degree dist. (female-female)	11.801	11.801	0	N/A
Global Clustering Coefficient (female)	0	0	0	N/A
Std. Dev. degree dist. (bipartite-female)	1.0171	1.0328	0.064	-0.244
Skewness degree dist. (bipartite-female)	1.5778	1.8488	0.287	-0.943
Std. Dev. degree dist. (bipartite-male)	0.9539	0.9883	0.054	-0.64
Skewness degree dist. (bipartite-male)	2.4407	3.1159	0.577	-1.171
Bipartite Clustering Coefficient	0.0169	0.0189	0.005	-0.409
Std. Dev. degree dist. (male-male)	0.083	0.083	0	N/A
Skewness degree dist. (male-male)	11.9484	11.9484	0	N/A
Global Clustering Coefficient (male-male)	0	0	0	N/A

References

[1] Wang P, Sharpe K, Robins GL, Pattison PE (2009) Exponential random graph (p*) models for affiliation networks. Social Networks 31: 12 - 25.

[2] Wang P, Pattison P, Robins G (2013) Exponential random graph model specifications for bipartite networks- a dependence hierarchy. Social Networks 35: 211 - 222.

[3] Wang P, Robins G, Pattison P, Lazega E (2013) Exponential random graph models for multilevel networks. Social Networks 35: 96 - 115.
